# Impact of microbiological molecular methodologies on adaptive sampling using nanopore sequencing in metagenomic studies

**DOI:** 10.1186/s40793-025-00704-7

**Published:** 2025-05-05

**Authors:** Josephine Herbert, Stanley Thompson, Angela H. Beckett, Samuel C. Robson

**Affiliations:** 1 Centre for Enzyme Innovation, University of Portsmouth, Portsmouth, Hampshire PO1 2DT UK; 2 Institute of Life Sciences and Healthcare, University of Portsmouth, Portsmouth, Hampshire PO1 2DT UK; 3 Department of Life Sciences, University of Bath, Bath, BA2 7AY UK

**Keywords:** DNA, Sequencing, Nanopore, Adaptive sampling, DNA extraction, Metagenomics, 16S rRNA, Third-generation sequencing

## Abstract

**Introduction:**

Metagenomics, the genomic analysis of all species present within a mixed population, is an important tool used for the exploration of microbiomes in clinical and environmental microbiology. Whilst the development of next-generation sequencing, and more recently third generation long-read approaches such as nanopore sequencing, have greatly advanced the study of metagenomics, recovery of unbiased material from microbial populations remains challenging. One promising advancement in genomic sequencing from Oxford Nanopore Technologies (ONT) is adaptive sampling, which enables real-time enrichment or depletion of target sequences. As sequencing technologies continue to develop, and advances such as adaptive sampling become common techniques within the microbiological toolkit, it is essential to evaluate the benefits of such advancements to metagenomic studies, and the impact of methodological choices on research outcomes.

**Aim and methods:**

Given the rapid development of sequencing tools and chemistry, this study aimed to demonstrate the impacts of choice of DNA extraction kit and sequencing chemistry on downstream metagenomic analyses. We first explored the quality and accuracy of 16S rRNA amplicon sequencing for DNA extracted from the ZymoBIOMICS Microbial Community Standard, using a range of commercially available DNA extraction kits to understand the effects of different kit biases on assessment of microbiome composition. We next compared the quality and accuracy of metagenomic analyses for two nanopore-based ligation chemistry kits with differing levels of base-calling error; the older and more error-prone (~ 97% accuracy) LSK109 chemistry, and newer more accurate (~ 99% accuracy) LSK112 Q20 + chemistry. Finally, we assessed the impact of the nanopore sequencing chemistry version on the output of the novel adaptive sampling approach for real-time enrichment of the genome for the yeast *Saccharomyces cerevisiae* from the microbial community.

**Results:**

Firstly, DNA extraction kit methodology impacted the composition of the yield, with mechanical bead-beating methodologies providing the least biased picture due to efficient lysis of Gram-positive microbes present in the community standard, with differences in bead-beating methodologies also producing variation in composition. Secondly, whilst use of the Q20 + nanopore sequencing kit chemistry improved the base-calling data quality, the resulting metagenomic assemblies were not significantly improved based on common metrics and assembly statistics. Most importantly, we demonstrated the effective application of adaptive sampling for enriching a low-abundance genome within a metagenomic sample. This resulted in a 5-7-fold increase in target enrichment compared to non-adaptive sequencing, despite a reduction in overall sequencing throughput due to strand-rejection processes. Interestingly, no significant differences in adaptive sampling enrichment efficiency were observed between the older and newer ONT sequencing chemistries, suggesting that adaptive sampling performs consistently across different library preparation kits.

**Conclusion:**

Our findings underscore the importance of selecting a DNA extraction methodology that minimises bias to ensure an accurate representation of microbial diversity in metagenomic studies. Additionally, despite the improved base-calling accuracy provided by newer Q20 + sequencing chemistry, we demonstrate that even older ONT sequencing chemistries can achieve reliable metagenomic sequencing results, enabling researchers to confidently use these approaches depending on their specific experimental needs. Critically, we highlight the significant potential of ONT’s adaptive sampling technology for targeted enrichment of specific genomes within metagenomic samples. This approach offers broad applicability for enriching target organisms or genetic elements (e.g., pathogens or plasmids) or depleting unwanted DNA (e.g., host DNA) in diverse sample types from environmental and clinical studies. However, researchers should carefully weigh the benefits of adaptive sampling against the potential trade-offs in sequencing throughput, particularly for low-abundance targets, where strand rejection can lead to pore blocking. These results provide valuable guidance for optimising adaptive sampling in metagenomic workflows to achieve specific research objectives.

**Supplementary Information:**

The online version contains supplementary material available at 10.1186/s40793-025-00704-7.

## Introduction

The development of next-generation sequencing (NGS) has greatly advanced the study of metagenomics, allowing for deeper and more-detailed investigations of microbial populations [66]. Indeed, NGS has become a common tool in a range of microbiological fields, including gut microbiome studies [5, 30], monitoring pathogen epidemiology [15, 48], and even the identification of novel plastic degrading enzymes for industrial scaling [20]. As tools such as these become common-place in molecular biology, it is important to assess the role played on downstream data analyses by methodological decision making and advances in technology to ensure optimal benefit.

Second generation NGS approaches, such as those from the market-leader and current gold standard Illumina, typically require fragmentation of target DNA prior to sequencing short fragments at high resolution [22]. More recently, third generation long-read sequencing technologies have been developed, most notably those from Pacific BioSciences and Oxford Nanopore Technologies (ONT). The nanopore-based system developed by ONT sequences DNA through thousands of ~ 1.4 nm pores implanted in a lipid bilayer with an ionic current flowing across them. DNA is unwound by a helicase enzyme attached to the sequencing adapter, and single-stranded DNA (ssDNA) is translocated through the pore. The resulting fluctuations in ionic flow across the membrane caused by the specific structure and polarity of each nucleotide are detected and converted into a DNA sequence by a machine learning classifier [3, 11, 61] (Figure S1).

In the past, nanopore-based base-calling has produced data with higher error rates than other NGS technologies, in particular as a result of misclassification of homopolymeric regions [12]. However, recent developments in the ligation chemistry used to prepare ONT libraries has resulted in the release of the Q20 + chemistry kit (SQK-LSK112, recently updated to SQK-LSK114) which promises improved average sequencing accuracy. The LSK112 chemistry claims to achieve simplex accuracies of over 99%, when combined with the R10.4.1 flowcell [34]. This comes as an improvement from the previous simplex accuracies achieved with LSK109 chemistry of ~ 97% with the R9.4.1 flowcell, showing significant improvements in data quality possible with updated chemistries, which has been found to have subsequent impacts on downstream data analyses [17, 57].

Nanopore sequencing allows for the end-to-end sequencing of extremely long DNA molecules, with a current record of 4.2 Mb achieved in-house by ONT (https://nanoporetech.com/about-us/news/blog-kilobases-whales-short-history-ultra-long-reads-and-high-throughput-genome; accessed 11th October 2023). Such long-read technologies provide the capability of bridging long repetitive regions of the genome with reads that span the entire region of repeats. These regions are typically difficult to resolve with short reads alone, so the use of long reads can aid genome completeness in de novo assembly, producing a more accurate and detailed whole-genome view [5]. Furthermore, the ability to resolve taxa to a species-level through assessment of the whole (or even partial) 16S ribosomal RNA (rRNA) gene provides increased resolution to community profiling analyses than can currently be achieved through short read sequencing of 16S rRNA hypervariable regions alone [5, 40]. Recently, the introduction of improved sequencing chemistry for ONT long read sequencing has demonstrated that high-quality contiguous metagenome assembled genomes (MAGs) can be produced using nanopore data alone, avoiding the need for genome polishing with higher quality short-read sequencing data [57].

The real-time base-calling approach employed by ONT also provides additional capabilities unique to this sequencing technology, including the recently developed adaptive sampling process first described by Loose et al. [33]. This approach allows the user to selectively enrich or deplete reads in real-time, based on whether they match customised sequences that may be of interest. This is implemented within the ONT sequencing software, MinKnow, by providing a database of target sequences as a FASTA file for enrichment or depletion. As the first few hundred nucleotides of the sequence are base-called, the software matches against the provided database, allowing a decision to be made regarding whether to continue sequencing to completion, or to reject the sequence and physically eject it from the pore [27, 50]. This approach has a wide range of potential applications, from detecting pathogenic genes in clinical samples [49], depleting host DNA in microbiome investigations [37], or enriching for rare species within metagenomic samples [38]. Multiple tools have been developed to facilitate adaptive sampling, employing different strategies for making real-time rejection decisions based on the first 160–450 base pairs of a read. These include dynamic time warping used by UNCALLED, read mapping approaches used by MinKNOW and ReadFish, and k-mer-based methods used by ReadBouncer [27, 50, 60]. A comparative study by Ulrich et al. [59] evaluated the efficacy of these tools and how they impact the adaptive sampling process, also demonstrating effective enrichment of bacterial plasmids. However, as ONT sequencing technologies continue to evolve rapidly, the potential biases introduced by updated sequencing technologies and their impact on adaptive sampling remain unclear, highlighting the need for further investigation.

Additionally, whilst such advancements provide a range of exciting possibilities for microbiological studies, initial unbiased extraction of DNA from metagenomic samples may prove challenging. For example, Gram-positive bacteria are notoriously difficult to lyse and are resistant to certain lytic enzymes, requiring specialised enzymatic mixtures such as MetaPolyzyme (Sigma-Aldrich, Missouri, USA, Product No.: MAC4L), or more physical lysing methods such as bead-beating [25, 43, 55, 63]. Such physical approaches, whilst resulting in more complete access to genetic material from the cells, may also result in increased fragmentation of the resulting DNA [51]. For sampling of microbial communities, it is therefore necessary to ensure that the DNA extraction method chosen is able to produce an unbiased picture of the diversity of microorganisms present, whilst also preserving DNA quality for downstream sequencing. This is particularly true for environmental samples, where diversity is often high [58].

This study aims to evaluate the effectiveness of adaptive sampling, a novel technique from ONT, in enriching low-abundance organisms within metagenomic samples, while providing critical benchmarks for methodologies that may influence its performance. This will help inform researchers using this powerful approach, and allow them to optimise experimental designs for metagenomic studies leveraging ONT sequencing. Using the well-established ZymoBIOMICS Microbial Community Standard (ZMC) [39], designed to assess biases in DNA isolation, we examine key factors that may influence adaptive sampling outcomes, including DNA extraction techniques and sequencing chemistry.

To contextualise adaptive sampling performance, we first analysed the quality and accuracy of 16S rRNA gene amplicon sequencing across a range of commercially available DNA extraction methods, identifying biases introduced by different kits. We then compared metagenomic assemblies generated using the older, more error-prone LSK109 sequencing chemistry and the newer, higher-accuracy LSK112 Q20 + chemistry from ONT. These comparisons provided valuable insights into how sequencing chemistry affects data quality, contiguity, and accuracy.

Finally, we employed adaptive sampling to enrich for the low-abundance yeast *Saccharomyces cerevisiae* within the mock community, assessing the impact of sequencing chemistry on real-time enrichment efficiency and genome completeness. This targeted approach allowed us to evaluate the practical applications of adaptive sampling for enriching specific taxa in metagenomic studies. Together, these findings aim to highlight key considerations for implementing adaptive sampling effectively and demonstrate its potential for advancing environmental and clinical microbiological research.

## Materials and methods

### DNA extraction

To compare the efficacy of different DNA extraction kits, five commercially available kits were selected from three companies (Table 1). These kits were chosen with cost, extraction methodology, and ease of use in mind. Extractions were performed following the manufacturer’s recommendations, with the exception of the AllPrep DNA/RNA Mini Kit (Qiagen, Germany, Product No.: 98204; AP), which was adapted for lysis of Gram-positive bacteria. The ZMC sample was first lysed in 100 µL of TE containing 15 mg/mL lysozyme and 4 mg/mL Proteinase K, was mixed by vortexing for 10 s, and further vortexed for at least 10 s every 2 min for 10 min. For the remainder of the protocol, the standard instructions were followed. For the Quick DNA/RNA MagBead Kit (Zymo Research Corporation, California, USA; Product No.: R2130; MB) the lysis for an environmental sample was followed within the recommended protocol. The ZMC sample was subjected to bead-beating using the FastPrep-24™ 5G bead beating grinder and lysis system (MP Biomedicals; Product No. 116005500) for 8.0 m/sec for 1 min. The remainder of the protocol was followed as per the manufacturer’s instructions.

**Table 1:** DNA extraction kits

Commercial kit	Abbreviation	Provider	Product no	Cost (RRP excludingVAT) (£)	Bead-beating	Extraction methodology
AllPrep DNA/RNA Mini Kit	AP	Qiagen	98,204	594.00	N	Column-based
RNeasy PowerSoil DNA Elution Kit	PS	Qiagen	12,866–25	497.00 (Including RNeasy PowerSoil Total RNA kit)	Y	Alcohol precipitation
QIAamp PowerFecal Pro DNA Kit	PF	Qiagen	51,804	352.00	Y	Column-based
FastDNA™ SPIN Kit	BM	MP Biomedicals	116,540,600-CF	405.00	Y	Column-based
Quick DNA/RNA™ MagBead	MB	Zymo Research	R2130	442.00	Y	Magnetic bead

Five commercially available DNA extraction kits were selected from three companies. Procurement information, cost (RRP excluding VAT), type of extraction method, and whether they employ a bead-beating technique are shown

Each DNA extraction was conducted on 75 µL ZymoBIOMICS Microbial Community Standard (Zymo Research Corporation, California, USA; Product No.: D6300; ZMC) in triplicate for each kit. For the FastDNA™ SPIN Kit (MP Biomedicals, California, USA; Product No.: 116540600-CF; BM), 250 µL of the ZMC was extracted in triplicate. The microbial standard is composed of ten microbial species, including eight bacteria and two yeast (Table S1). The standard contains ~ 1.4 × 10^10^ cells mL^−1^ in total, and the organisms are distributed equally in theoretical genomic DNA composition at 12% each for bacteria, and 2% each for yeast species.

### DNA quality control

Assessment of 260/230 and 260/280 ratios was performed to determine DNA quality and purity using the DS-11 Series Spectrophotometer (DeNovix Inc., Delaware, USA), with DNA concentration estimated using Qubit fluorometric quantification (Thermofisher Scientific™, Massachusetts, USA) (Table S2). Each sample was also evaluated using the 4150 Tapestation (Genomic DNA Assay; Agilent Technologies, Inc., California, USA; Product No.s: 5067–5365, 5067–5366) to assess the impact of extraction methodology on fragment length and also to calculate optimal fentimolar input for the metagenomic sequencing library preparation (Figure S2).

### 16S rRNA amplification

PCR amplification of the entire 16S rRNA gene was conducted on DNA extractions from ZMC samples for the kit comparison using the following primers: forward primer (5’-AGAGTTTGATCMTGGCTCAG-3’), reverse primer (5’-ACGGYTACCTTGTTACGACTT-3’). The PCR reaction contained 25 µL volumes containing 12.5 µL ReadyMix™ Taq PCR Reaction Mix (Sigma-Aldrich, Missouri, USA; Product No.: P4600) or Q5 High-Fidelity 2X Master Mix (New England BioLabs, Massachusetts, USA; Product No.: M0492S), 0.5 µL forward and 0.5 µL reverse primers (100 µM), and 0.5 µL extracted total DNA, performed using the SimpliAmp™ Thermal Cycler (ThermoFisher Scientific™). The PCR was performed with an initial denaturation for 3 min at 94 °C followed by 40 cycles of denaturation for 45 s at 94 °C, primer annealing for 60 s at 57 °C and polymerase extension for 120 s at 72 °C, followed by a final primer extension step for 7 min at 72 °C.

PCR product was confirmed using gel electrophoresis (0.8% agarose, 90 V, 55 min) (Figure S3) and quantified by Qubit fluorometric quantification using the dsDNA High Sensitivity Assay (Thermofisher Scientific™ Massachusetts, USA; Product No.: Q32851). Subsequently, PCR product was purified for use in ONT sequencing using the QiaQuick PCR Purification Kit (Qiagen; Product No.: 28104).

### Sequencing

ONT sequencing libraries were generated from amplified PCR products or metagenomic extractions of the ZMC sample using the ligation sequencing kit by ONT (either SQK-LSK109 or SQK-LSK112) and native barcoding kits (either EXP-NBD104 and EXP-NBD114 or SQK-NBD112.24, respectively). Libraries were created as per the manufacturer’s instructions, with one minor modification; no FFPE DNA repair mixture was used in Master Mix 1 for LSK109 library preparation for the 16S rRNA amplicon library, as recommended for the LoCost SARS-CoV-2 amplicon protocol (https://www.protocols.io/view/ncov-2019-sequencing-protocol-v3-locost-bp2l6n26rgqe/v3; accessed 11th January 2023).

Libraries were loaded onto either R9.4.1 flowcells for LSK109 chemistry, or R10.4.1 flowcells for LSK112 prepared libraries. Super Accuracy base-calling (Guppy v6.1.5) was employed for both the LSK109 and LSK112 sequencing libraries through the ONT MinKnow software v22.05.7.

### Adaptive sampling

For the library prepared with the LSK109 chemistry, after 8 h of full genomic sequencing, the run was stopped and restarted with adaptive sampling enabled, targeting the entire *S. cerevisiae* R64 genome from the National Centre for Biotechnology Information (NCBI) assembly database (https://www.ncbi.nlm.nih.gov/assembly; RefSeq accession GCF_000146045.2) using MinKNOW software (ONT) using default parameters. Chosen due to its low abundance of only 2% genomic DNA composition within the ZMC, which is desirable when selecting for an organism to enrich for [38]. Currently, there are no options to modify the adaptive sampling approach to enable for more leniency in sequence acceptance for example, although this may change in the future. For the LSK112 prepared library, the same approach was used with adaptive sampling initiated after 8 h of full genomic sequencing. For adaptive sampling, the Fast base-calling algorithm was used at run-time to improve adaptive sampling efficiency, but samples were re-base-called using Super Accuracy (Guppy v6.1.5) prior to downstream data analysis. Both LSK109 and LSK112 adaptive sampling runs were sequenced for 72 h.

### 16S rRNA amplicon sequencing analysis

For analysis of 16S rRNA gene amplicon sequence data, the raw sequencing data were first assessed for quality using Nanostat v1.6.0 and MinIONQC v1.4.1 [28]. Reads were then adapter-trimmed using Porechop v0.2.4 [62] and filtered to discard reads with a quality score lower than Q10 and reads not between 1.2–1.8 Kb in length using NanoFilt v2.8.0 [7]. Emu v3.4.5 was used for taxonomic assignment, employing an expectation–maximization algorithm for taxonomic assignment of long-read 16S rRNA gene amplicon sequences [8], using parameters ‘–type map-ont –min-abundance 0.0001 -N 50 –keep-counts’. Taxa were determined through mapping to the SILVA v138.1 (Quast et al*.*, 2013) database, using the pre-built Emu v3.0 + database generated from the DADA2 SILVA species-level database.

Taxa annotated reads were analysed further using R v4.1.2, including *Phyloseq* v1.38.0 [41] for figure generation, *Ape* v5.6–2, [47] for phylogenetic tree construction, and *Vegan* v2.6–4 [44] for multivariate analyses. Due to uneven read depths between kits due to biases during sequencing, the samples containing fewer than 20,000 reads (PF_1 and PS_3) were removed as outliers. Following this, remaining samples were rarefied to the sample with the lowest read count (Figure S4; PS_1; 26,903) to ensure an even sampling depth for the comparison (Table S3).

### Metagenomic assembly analysis

For whole genome metagenomic data, to assess the proportion of reads mapped to each expected microbe within the ZMC, the samples were aligned to the non-redundant nt database (including annotations from GenBank, Refseq, Third Party Annotation, and Protein Database sequences) using Kraken2 v2.1.3 [64]. This database was the most recently available at the time (29/11/2023), available from the official Kraken2 index site (https://benlangmead.github.io/aws-indexes/k2). The data were also mapped to a database consisting of the whole genomes of each strain contained within the ZMC based on information obtained from Zymo Research Corporation ([67], Table S1) using BLAST v2.12.0 + [1, 6] to ascertain the read lengths for each organism (Figure S6).

To assess genome assembly quality, raw reads were filtered to remove poor quality and short reads (Q = < 7, < 500 bp) in the same manner as described above. The reads were mapped to each reference from the expected bacteria within the ZMC using minimap2 v2.22 [31]. The resulting mapped reads were converted from a bam to fastq file, which was then subsampled to even coverage between the sequencing chemistries using seqtk [32]. Assemblies were then generated for each bacterial genome using flye v2.9.1 using the nano-hq flag with default parameters [26]. The assembly statistics of these assemblies were assessed using Quast v5.2.0 [19], BUSCO v0.4.5 [36] and the *pafr* package (https://github.com/dwinter/pafr; accessed 15th January 2022; v0.0.2) in R v4.2.3 [54] for alignment visualisation.

### Adaptive sampling enrichment analysis

For the adaptive sampling runs, reads were trimmed to remove adapters, poor-quality, and short reads (Q = < 7; read length < 1000 bp). In addition, reads were further trimmed to retain only those classified as passing the adaptive sampling algorithm (identified by a decision entry of “stop_receiving” in the adaptive sampling report) using seqtk v1.4. Reads were mapped against a database containing whole genome sequences from the NCBI database for species within the ZMC sample (https://www.ncbi.nlm.nih.gov/assembly; accession number for *S. cerevisiae*: GCF_000146045.2 and [67] using BLAST v2.12.0 + . This organism represents 2% of the overall genomic DNA composition (cell number = 0.29%) within the ZMC (Table S1), and was selected as the target due to having an on-target abundance that is less than 10% (which is suggested by ONT to provide maximum benefit,https://nanoporetech.com/document/adaptive-sampling; accessed 28th October 2024), and similar to previous studies that have investigated the efficacy of adaptive sampling [38].

To assess whether the reads enriched through adaptive sampling covered the whole *S. cerevisiae* genome, the reads were first mapped against the reference using minimap2 v2.22. The resulting mapping file was then investigated for depth and coverage using samtools v1.18 and bedtools v2.29.2 [10, 53] and plotted using the *circlize* package (v0.4.16) in R for visualisation [18, 54].

To investigate the enrichment of *S. cerevisiae*, the adaptive sampling fastq files were firstly subsampled to the first 8 h run to match the corresponding metagenomic datasets using seqtk v1.4. To normalise data for comparison between datasets and to account for biases due to standard read rejection during the process of adaptive sampling, only bases mapped to *S. cerevisiae* were considered. To calculate the number of bases mapped to *S. cerevisiae* for each sample, the fastq files were first mapped to the *S. cerevisiae* reference genome (Accession Number: GCF_000146045.2) using minimap2 v2.22. Samtools v1.18 was used to filter reads to remove unmapped reads (-F 4), secondary alignments (-F 256), and supplementary alignments (-F 2048). Finally, GNU Awk v5.0.1 was used to calculate the total length from the resulting unique reads giving the total number of bases. To calculate enrichment, the number of nucleotides mapping to *S. cerevisiae* after all processing was completed (i.e. after all trimming steps) were divided by the total number of nucleotides within the raw data for each replicate, then multiplied by 100 for percentage. This gives the percentage of all nucleotides generated by the sequencing run that map to the *S. cerevisiae* genome for each sample, which could then be compared between adaptive and non-adaptive runs.

### Statistics

Before performing statistical tests for significance, data were tested for normality using the Shapiro-Wilks test using the *shapiro.test()* function from the *stats* package (v3.6.2) in R. If data were found to be normal, paired *t*-test was performed with Benjamini–Hochberg multiple testing correction using the *t_test()* function. For non-parametric variables, a paired Wilcoxon signed-rank sum test with Benjamini–Hochberg multiple testing correction was used using the *wilcox_test()* function to test for statistical differences between groups. For analysis of differences in the counts between taxa across the populations, Chi-squared analysis was performed using the *chisq.test()* function, and Bray–Curtis dissimilarity was assessed using the *vegdist()* function. Correlations between data were analysed using Spearman’s rank correlation coefficient using the *cor.test()* function.

## Results

### DNA extraction kits produce variable estimates of diversity

**Fig. 1:**
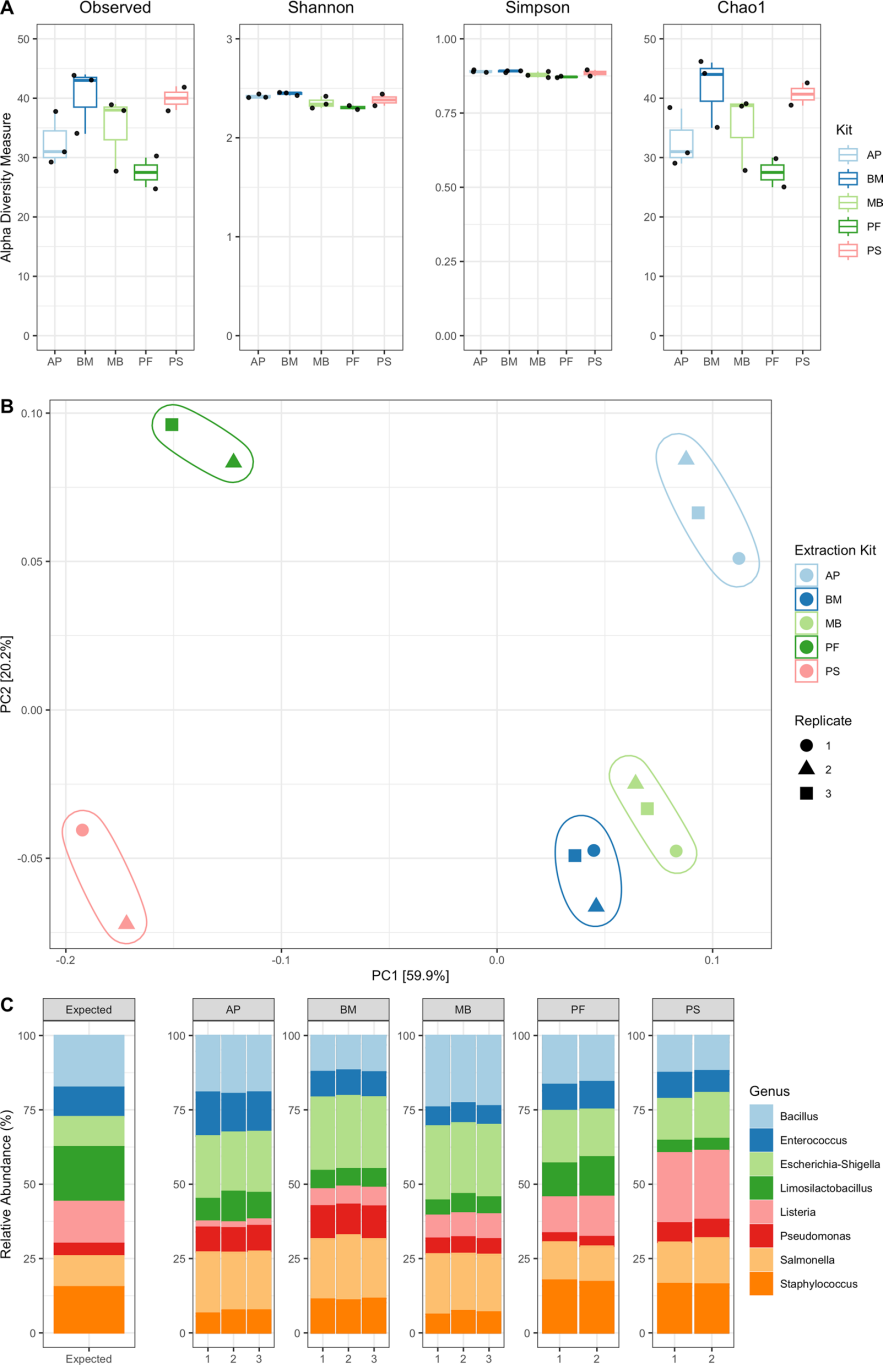
16S rRNA amplicon sequencing diversity analyses between commercial DNA extraction kits. **A** Species-level alpha diversity of the samples from each DNA extraction kit (following rarefaction) across the dataset, represented by observed Shannon, Simpson, and Chao1 indices. Analysis between groups shown using paired t-test with Benjamini–Hochberg multiple testing correction (after testing for normality using a Shapiro-Wilks test). **B** Species-level principal coordinate analysis (PCoA) of the samples from each DNA extraction kit across the dataset. **C** Relative abundance of expected 16S rRNA gene within the ZMC for each genus, and the 16S rRNA gene amplicon relative abundances achieved using each DNA extraction kit. The genus abundance was ascertained through the use of 16S rRNA gene amplicon sequencing using the LSK112 kit

For comparison of the different DNA extraction kits, alpha diversity was investigated to observe evidence of systematic bias in the diversity of bacteria extracted using the different methods. Four measures of alpha diversity were assessed: observed diversity, Shannon diversity index, Simpson diversity index and Chao1 diversity index (Fig. 1A). When comparing indices pairwise between the kits, the difference in the means of the Observed, Shannon, Simpson, and Chao1 indices were all insignificant, suggesting that all kits produce similar diversity metrics. Together with the similarity in the throughput for the replicates (Table S3), this suggests that these kits show high reproducibility. However, it should be noted that similarity in alpha diversity may be unsurprising given the sample consisted of only eight bacteria and so these results may not be generalizable to more diverse populations.

Beta diversity was therefore investigated using principal coordinate analysis (PCoA) with Bray–Curtis dissimilarity, to explore overall variance between kits (Fig. 1B). This analysis revealed clear clustering of samples by extraction kit, suggesting that within group variation was lower than between group variation. Indeed, separation between the different kits within the first two principal coordinates (PC) accounted for 80.1% of the total variance in the data. This is supported by PERMANOVA analysis which revealed significant differences between the kits (*p* value = 0.001, *df* = 4, *f* = 97.39, *R*^*2*^ = 0.98). These suggest variance in the resulting composition of the ZMC depending on the DNA extraction kit used, signifying kit-specific biases. Variance on PC1 seems to cluster kits based on methodology of extraction, with BM and MB clustering very closely having undergone the same bead-beating process separate from the three other kits which all had different bead-beating (PS, PF) or enzymatic lysis (AP) methodologies. Although, this may not entirely be a separation based on whether bead-beating was employed as AP clusters more closely to BM and MB despite not having undergone beat-beating. PF and PS appear the most dissimilar kits to the others, with separation of PS possibly explained by the high levels of degradation seen within the DNA (Figure S2). However, this does not explain the separation of PF which has comparatively intact DNA.

These results agree with previous studies highlighting that the methodology employed by each extraction kit may result in a characteristic composition of taxa [14, 24, 25, 55]. To fully explore this, we identified the abundance of taxa identified for each sample (Fig. 1C) and compared this with the expected abundance from the ZMC using Chi-squared analyses. This identified all the kits as significantly different from the expected distribution (AP: *X*^*2*^ = 6168, *df* = 7, *p *value = < 2.2e-16; BM: *X*^*2*^ = 5893, *df* = 7, *p *value = < 2.2e-16; MB: *X*^*2*^ = 5622, *df* = 7, *p* value = < 2.2e-16; PF: *X*^*2*^ = 796, *df* = 7, *p *value = < 2.2e-16; PS: *X*^*2*^ = 3788, *df* = 7, *p *value = < 2.2e-16). This is likely due to the overrepresentation of Gram-negative genera including *Escherichia-Shigella* (Expected: 10.1%; AP: 20.2%; BM: 24.0%; PF: 16.3%; PS: 14.6%; MB: 24.2%) and *Salmonella* (Expected: 10.4%; AP: 20.2%; BM: 20.4%; PF: 12.6%; PS: 14.5%; MB: 19.3%) seen in all kits. The Gram-positive genus *Limosilactobacillus* genus was particularly underrepresented in all kits (Expected: 18.4%; AP: 8.7%; BM: 5.9%; PF: 12.3%; PS: 4.0%; MB: 5.7%). There are also discrepancies that are more unique to each kit, with considerable underrepresentation of *Listeria* in the AP and BM kits (Expected: 14.1%; AP: 2.0%; BM: 6.01%) but overrepresentation seen in the PS kit (23.3%).

These data highlight that the chosen DNA extraction kit introduces biases to the extracted community in such a way that the diversity may not be truly representative of the sample, with some kits potentially more suitable depending on the context. From these analyses, the MB and BM kits appeared to produce the least biased representation for the ZMC when comparing the kits to one another, although all showed statistically significant differences in composition to the expected community profile. When assessing the visual composition of the microbial composition obtained from the kits (Fig. 1C) and considering the expected percentages of each genus as described above, PF appeared most closely similar to the expected abundance and showed the lowest Chi-squared statistic of all kits. This is also supported by Bray–Curtis dissimilarity analysis which was performed on the counts of expected species, which showed PF to show the least dissimilarity to the expected composition (BC = 0.1003), followed by the PS kit (BC = 0.2200) and then AP (BC = 0.3048), MB (BC = 0.3088) and finally BM (BC = 0.3100).

### Sequencing chemistry does not influence metagenomic abundance profiles

**Fig. 2:**
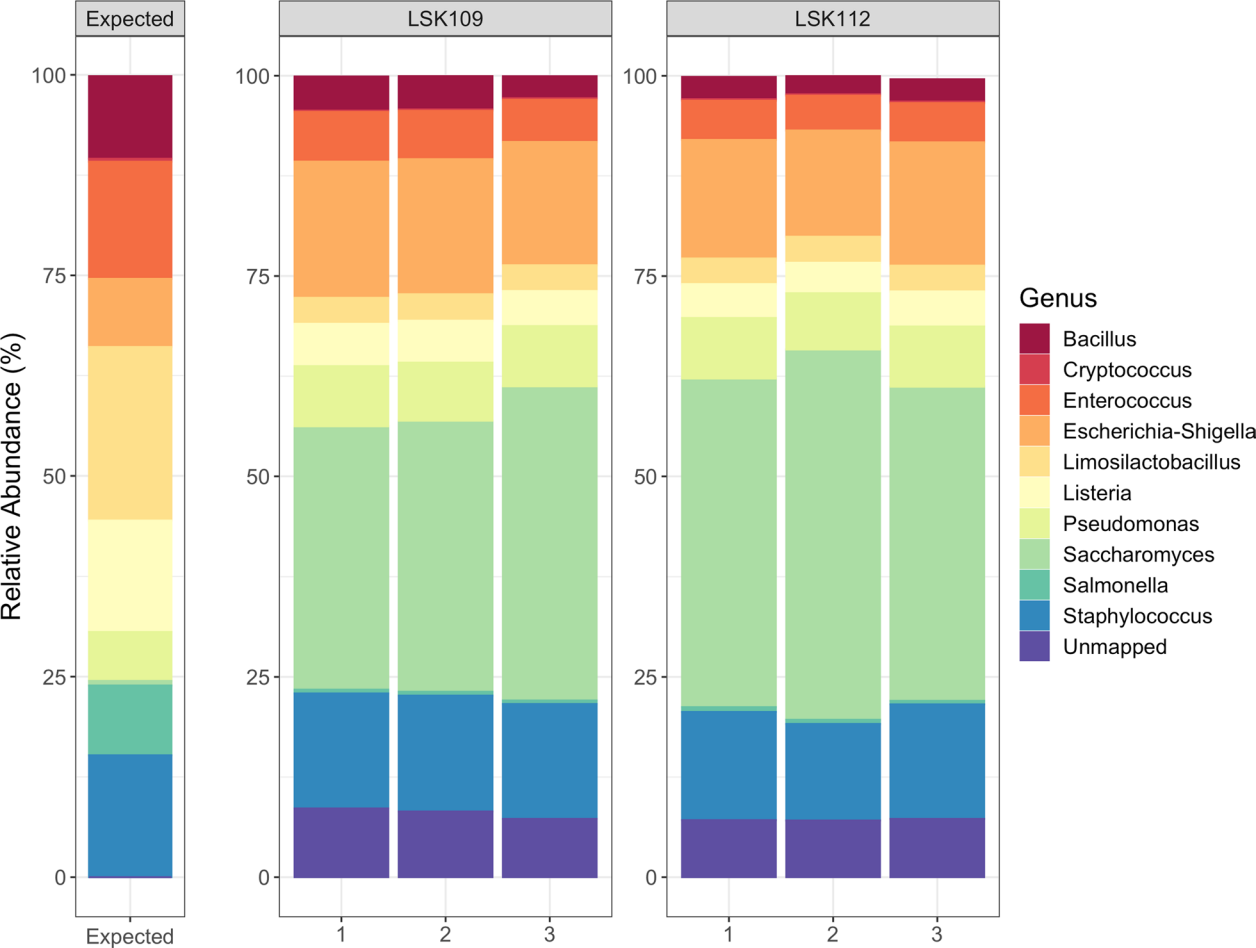
Metagenomic comparison between ONT sequencing chemistries. Relative abundance of the number of reads mapped to each expected organism within the ZMC, from DNA extracted using the BM kit and sequenced using both the LSK109 and Q20 + LSK112 chemistries. Results are shown for whole metagenomic sequencing. Left panel shows expected genomic composition (estimated genome copy number) of each organism within the ZMC Standard

To explore the role played by sequencing chemistry and base-calling quality on the accuracy of metagenomic assembly data, ZMC whole metagenomic extractions using the BM kit were sequenced using both the older, more error-prone LSK109, and the more recent Q20 + LSK112 chemistry library kits (Fig. 2; Table S4). No significant difference between the two sequencing chemistries was identified when examining the percentage of reads obtained for each organism within the ZMC (*X*^*2*^ = 0.05, *df* = 10, *p *value = 1). However, both showed a significant difference in the composition compared to the expected distribution (LSK109: *X*^*2*^ = 60.01, *df* = 10, *p *value = 3.60e-09; LSK112: *X*^*2*^ = 71.79, *df* = 10, *p *value = 1.99e-11). This is predominantly due to a considerable overrepresentation of *Pseudomonas* (LSK109: 31.95 ± 2.40%; LSK112; 42.11 ± 3.64%; Expected: 6.1%), and lower than expected values of certain Gram-positive genera such as *Limosilactobacillus* (LSK109: 3.11 ± 0.28%; LSK112: 3.22 ± 0.02%; Expected: 13.9%) and *Listeria* (LSK109: 5.53 ± 0.46%; LSK112; 4.14 ± 0.29%: Expected; 13.9%)*.* There are also higher than expected levels of ‘other’ organisms within the compositions (LSK109: 5.53 ± 0.15%; LSK112: 5.21 ± 0.08%), these however mostly belong to highly related species of those within the ZMC (e.g. reads were sometimes classified as *Bacillus spizizenii* instead of *B. subtillis*).

### *Q20* + *ONT chemistry increases assembly accuracy with minimal impact on genome completeness from metagenomic samples*

**Fig. 3:**
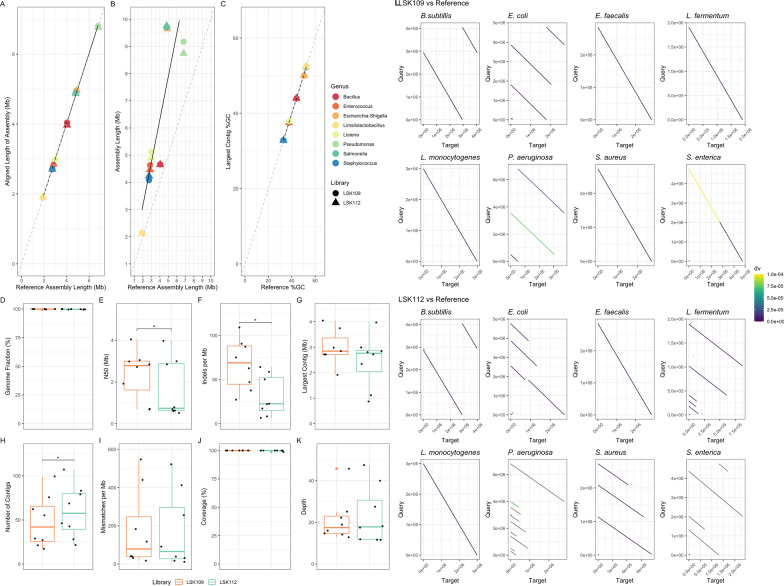
Metagenomic assembly statistics comparison between ONT sequencing chemistries. Comparison of whole genome de novo assemblies from metagenomic sequencing of the ZMC. Genome quality is assessed based on metrics obtained using Quast and Samtools, and alignments to the Zymo Research Corporation reference genomes. **A** Length of aligned assemblies against respective reference assemblies (line of best fit to the data shown in black and x = y line shown as a dotted grey line). **B** Total length of assemblies against respective reference assemblies (line of best fit to the data shown in black and x = y line shown as a dotted grey line). **C** GC content (%) of the largest contig of the assemblies against that of the reference genomes (line of best fit to the data shown in black and x = y line shown as a dotted grey line). **D** Genome fraction (%) of the assemblies to the references. **E**) N50 values of each ZMC assembly (Mb). **F** Number of insertions and deletions (indels) for each ZMC assembly per Mb. **G** Largest contig within each ZMC assembly (Mb). **H**) Number of contigs within each ZMC assembly. **I** Base mismatches within each ZMC assembly per Mb. **J** Coverage of reads (percentage of bases covered) to each ZMC assembly (%). **K** Depth of reads (mean depth of coverage) for each ZMC assembly. Comparison between chemistries is based on a paired Wilcoxon Signed rank sum test with Benjamini–Hochberg multiple testing correction (* = < 0.05). **L**) Dotplots of the assembled genomes of each expected bacterial species within the ZMC. Genomes assembled from LSK109 and LSK112 reads, aligned by divergence (dv; approximate per-base difference) between the query (reference genome) and target (genome assembled within study)

To assess whether the use of the less error prone Q20 + LSK112 sequencing chemistry from ONT impacted the quality and completeness of whole genome metagenomic assemblies, we compared a range of metrics between the de novo assemblies and their published genome counterparts (Fig. 3, Figure S5, and Table S5).

Despite showing generally larger overall lengths than the reference (Fig. 3A), the resulting largest contig of each assembly for each species showed significantly correlated GC content to the references used (Fig. 3B; 95% confidence; *Spearman’s rank correlation coefficient:* 0.99,* t* = 207.79, *df* = 14, *p *value < 2.2e-16), with significantly correlated alignment lengths (Fig. 3C; 95% confidence; *Spearman’s rank correlation coefficient*: 0.99, *t* = 130.48, *df* = 14, *p *value < 2.2e-16). Additionally, values obtained from genome quality assessment using Quast and Samtools (Fig. 3D–K and Table S5) suggest high-quality metrics for de novo assemblies for both chemistry data sets. Similarly, assessment using BUSCO revealed all assemblies to contain almost all marker genes expected (98.4–100%), suggesting that these represented complete assemblies.

Variation between the assemblies for the LSK109 and LSK112 chemistries can be seen, most notably with a significant decrease in the number of indels per Mb (Fig. 3F; *Wilcoxon signed-rank sum test, W* = 35*, p *value = 0.02) in assemblies created using the Q20 + LSK112 chemistry compared to the more error-prone LSK109 chemistry. However, interestingly there was no significant difference between mismatches per Mb (Fig. 3I; *Wilcoxon signed-rank sum test, W* = 26*, p *value = 0.31), despite the overall difference in the base-calling quality between the two data sets (Table S4). Interestingly, assemblies from the LSK112 data set showed a lower N50 (Fig. 3E; *Wilcoxon signed-rank sum test, W* = 33*, p *value = 0.004) and an increased number of contigs (Fig. 3H; *Wilcoxon signed-rank sum test, W* = 0*, p *value = 0.014) across the assemblies, which is seen equally for all organisms (Table S5). This indicates that more fragmented assemblies were seen within the LSK112 libraries for this sequencing run, despite matching read numbers. However, no significant differences were detected between the sequencing chemistries for other genome assembly quality metrics (genome fraction: *Wilcoxon signed-rank sum test, W* = 25*, p *value = 0.33; largest contigs: *Wilcoxon signed-rank sum test, W* = 30*, p *value = 0.109; coverage: *Wilcoxon signed-rank sum test, W* = 15*, p *value = 0.06; depth: *Wilcoxon signed-rank sum test, W* = 21*, p *value = 0.74).

The assemblies were further investigated by using dotplots to compare the assemblies against the existing reference genomes (Fig. 3L), and against one another (Figure S5). These plots are broadly similar between the sequencing chemistries, with similar alignments and no one chemistry performing significantly better than the other. However, as previously mentioned, LSK109 appears to result in less fragmented assemblies in many of the species. High divergence (approximate per-base difference) between the reference and assemblies within this study can be seen in some instances, such as in the assembly of *L. fermentum* using LSK109 and *L. monocytogenes* using LSK112. Thus, this effect is not necessarily chemistry specific, although it does appear that LSK112 suffers less from divergence in its assemblies, which is reflected in the number of indels per Mb.

### Adaptive sampling effectively enriches complete low abundance genomes from metagenomic samples

**Fig. 4:**
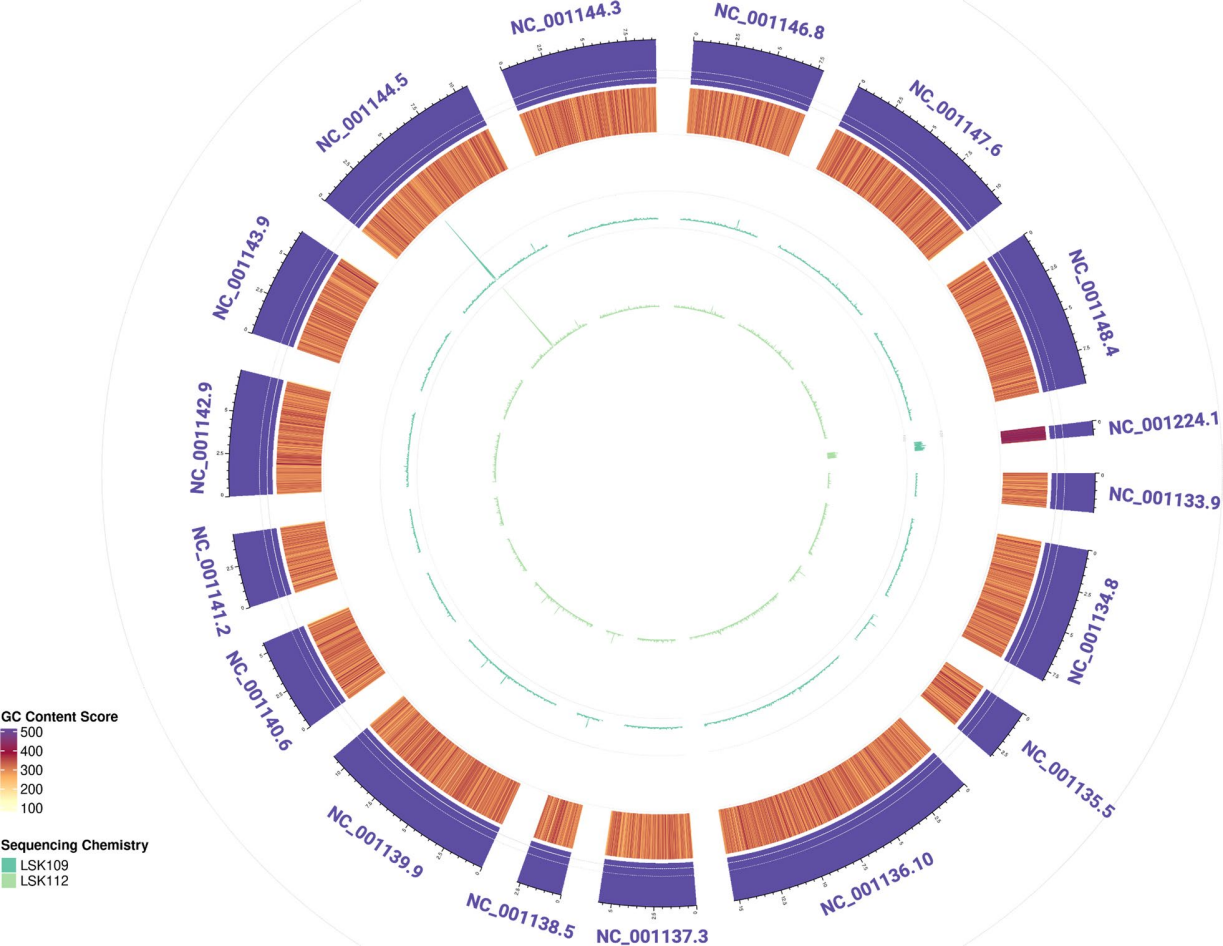
Genome completeness for adaptive sampling enriched *S. cerevisiae*. Circos plot showing the mapping of reads enriched for *S. cerevisiae* using adaptive sampling against the reference genome to assess differences in coverage between the LSK109 and LSK112 library preparation methods. Outer track shows the reference assembly of *S. cerevisiae* (Accession No.: GCF_000146045.2), with the inner track showing a heatmap of GC content score calculated using bedtools nuc, and the next two inner tracks in green, the depth of reads covering each region using LSK109 or LSK112 sequencing chemistry

To assess the effectiveness, accuracy, and completeness of the ONT-specific adaptive sampling mode for real-time enrichment of nanopore reads, adaptive sampling was used to specifically enrich for *S. cerevisiae from* whole genome metagenomic sequencing of the ZMC (Fig. 4)*.* Standard whole genome metagenomic sequencing using the LSK109 chemistry identified 0.29 ± 0.06% of quality trimmed bases mapping to *S. cerevisiae* (Table S4), compared to the expected 0.57% (theoretical genome copy number composition). Following adaptive sampling enrichment, this increased to 1.98 ± 0.28%. Similarly, for the LSK112 chemistry, non-adaptive sampling sequencing identified around 0.37 ± 0.03% of reads mapping to *S. cerevisiae*. Following adaptive sampling and filtering, this increased to 1.79 ± 0.26% bases mapping to *S. cerevisiae*. Enrichment in the proportion of bases mapping to *S. cerevisiae* between adaptive and non-adaptive sampling runs thus resulted in enrichments of 4.8-fold and 6.8-fold respectively for LSK109 and LSK112 sequencing chemistries. This matches estimates from ONT themselves, and from findings from other similar studies of enriched microbial whole genomes [38, 42]. Enrichment in the proportion of bases mapping to *S. cerevisiae* between non-adaptive runs and adaptive sampling runs was statistically significant for both sequencing chemistries (LSK109: *paired t-test*, *t* = 9.86, *df* = 2, *p *value = 0.008; LSK112: *paired t-test*, *t* = 9.04, *df* = 3*, p *value = 0.011), although the LSK112 kit indicated slightly greater enrichment.

It is worth noting that, despite this enrichment in the proportion of on-target nucleotides sequenced, the use of adaptive sampling in our hands resulted in a decrease in the total yield of data within 8 h for adaptive sampling (LSK109: 329.02 ± 371.9 Mbp; LSK112: 151.20 ± 147.7 Mbp) compared to the non-adaptive libraries (LSK109: 902.55 ± 1002.0 Mbp; LSK112: 557.92 ± 520.3 Mbp). Despite this reduction in throughput, the final real-term yield of data mapping to the target *S. cerevisiae* genome for LSK109 was greater for adaptive sampling (6.14 ± 7.1 Mbp) compared to the non-adaptive libraries (2.49 ± 2.9 Mbp; Table S4). For LSK112, only a marginal increase in overall yield was seen for adaptive sampling (2.48 ± 2.4 Mbp) compared to non-adaptive sequencing (2.09 ± 2.1 Mbp). This may relate to the increased fragmentation of DNA seen in our LSK112 library compared to the LSK109 library, which can have an impact on pore health and data throughput. Thus, it is important to optimise the sequencing runs in order to maximise the benefits of using adaptive sampling.

We next investigated whether the coverage of the *S. cerevisiae* genome from adaptive sampling resulted in equal coverage across the whole genome, or whether certain locations were preferentially enriched. Analysis on the combined replicate dataset showed that reads enriched for *S. cerevisiae* covered the genome equally for LSK109 sequencing chemistries, with average coverage over all chromosomes of 90.41 ± 3.62% and a read depth of 4.05 ± 4.35 (Table S6). The coverage for LSK112 was much lower at 55.11 ± 11.35%, although this is likely due to lower read throughput (Table S4) reflected in the lower depth of reads across the genome (1.15 ± 1.37). Enrichment of reads appears consistent between the two chemistries, with significantly higher depth seen mapping consistently to chromosome XII (Accession No.: NC_001144.5) and chromosome MT (Accession No.: NC_001224.1) for both samples (Fig. 4). Both loci contain rRNA genes with high levels of gene copies present, resulting in peaks within these regions of high copy number [21].

## Discussion

Whole metagenome sequencing and 16S rRNA gene amplicon-based profiling of microbial community samples are important tools for microbiology and are popular due to improvements in throughput, quality, ease of use, and reduction in associated costs. Additionally, novel techniques introduced by ONT, such as adaptive sampling, offer exciting opportunities to enhance specific microbiological studies. However, before implementing such approaches, it is crucial to investigate potential sources of bias that could influence the results. These biases may result from factors such as the choice of ONT sequencing chemistries, which have been shown in previous studies to impact data outcomes [17, 57]. The real-time adaptive sampling approach available when using ONT nanopore based sequencing technologies has demonstrated a number of potential applications within microbiological studies, including rapid diagnosis of infectious diseases, enrichment of plasmids, or depletion of host DNA in studies of microbiomes [37, 38, 59]. This approach requires an initial step whereby base-called reads are compared in real time against a known database for either enrichment or depletion [38]. In particular, to ensure the maximum length of sequence generated at the point of comparison with the relevant database, fast base-calling models are used, which show lower quality than other slower but higher accuracy models. Base-calling quality may therefore impact the identification of reads for enrichment/depletion in this process, particularly when high sequence similarity may exist between reads. We therefore explored whether adaptive sampling selected evenly across the genome when enriching for a single species within a metagenomic sample, and importantly whether the adaptive sampling approach was impacted by the use of an older sequencing chemistry with lower accuracy (~ 97%) compared to the newer Q20 + chemistry (~ 99%). Interestingly, when applying adaptive sampling to these metagenomic samples, minimal differences were seen in enrichment between the LSK109 and LSK112 sequencing chemistries, with both showing significant specific enrichment for bases mapping to *S. cerevisae.* The sequencing enrichment for whole genome (LSK109: 4.8-fold; LSK112: 6.8-fold) is in line with what is expected from current standards of adaptive sequencing for a low abundance organism [42], in particular Martin et al. [38] who were able to enrich low abundance organisms (~ 2%) by almost 5x. The reads that mapped to the *S. cerevisiae* genome with adaptive sampling produced reasonable coverage (percentage of bases covered) with low read depth (depth of covered bases) across the genome (Fig. 4; LSK109: coverage, 90.41 ± 3.62%; depth, 4.05 ± 4.35; LSK112: coverage, 55.11 ± 11.35%; depth, 1.15 ± 1.37; Table S6). These suggest an even enrichment across the whole genome, with higher coverage for LSK109 chemistry compared to LSK112, with regions containing rRNA genes showing greater enrichment due to higher copy numbers. Whilst coverage of the genome was almost complete for LSK109, the read depth was low for most regions, particularly for the comparatively low coverage LSK112 samples. This could be improved through increasing the depth of sequencing, or by targeting a species of interest whose genome represents a larger on-target region of interest amongst those present in the community. However, adaptive sampling is recommended for enrichment of species present at less than 10%, as there is a practical limit to the benefits that this approach might offer beyond this.

For both chemistries, it is important to note that adaptive sampling resulted in much lower throughput in real terms of the number of reads generated (Table S4). This has previously been seen in other studies, which has been theorised to be due to fewer actively sequencing pores due to rejection and repeat capture of reads [38, 50, 59]. This has been confirmed by ONT, who state “adaptive sampling runs are more likely to block pores due to the high amount of strand rejection”, which can be counteracted by the use of short reads or loading more sample than usual (https://nanoporetech.com/document/adaptive-sampling; accessed 28th October 2024). These results suggest that, despite the differences in base-calling error rates, sequencing chemistry likely makes little difference for enrichment yield through adaptive sampling. Our observed effects on coverage and data depth are likely influenced by sequencing depth, which may be reduced due to the lower overall throughput seen when adaptive sampling was enabled. Additionally, the lower quality LSK112 data may be further impacted by flow cell variability, as observed in the metagenomic data. Thus, for enrichment of low abundance genomes of interest within a metagenomic community, adaptive sampling represents a viable option for obtaining good quality whole genome data. For optimal results, the impacts on overall throughput of data should first be considered, and the sample for enrichment should consist of shorter reads to counteract impacts on pore health. However, this is currently being addressed by ONT with the release of updated versions of the MinKnow software which, since performing our experiment, now offer “ faster rejection time and better classification of reads to be rejected” (V24.06.10; https://community.nanoporetech.com/posts/software-release-24-06-10-14593; accessed 13th January 2025) and more recently “improved Adaptive Sampling and concurrent live base-calling using the option to only base-call on-target reads” (V24.11.8; https://community.nanoporetech.com/posts/software-release-24-11-8-f; accessed 16th January 2025). Additionally, choice of adaptive sampling software has the potential to affect yield, as shown by Ulrich et al. [59] when enriching for bacterial plasmids by depleting chromosomal elements of known bacteria.

For whole genome sequencing, the metagenomic assemblies in general revealed few differences between the sequencing chemistries (Fig. 3). Both sequencing chemistries produced complete genomes based on the presence of expected BUSCOs and alignment analysis. However, these may not be completely accurate for some species based on the dotplot analysis (Fig. 3L), with some assemblies showing high divergence (approximate per-base divergence) or even no data (including *E. faecalis*, *L. fermentum*, *L. monocytogenes, P. aeruginosa* and *S. aureus*), or producing larger than expected assemblies compared to the references (Fig. 3B). This may be due to the presence of contigs that may not map well to each assembly and yet were neither filtered nor excluded, or the metagenomic nature of the data, which notoriously makes robust and accurate assembly more difficult [29]. Thus, in this context, the accuracy of the base-calling for the sequencing chemistry may surprisingly make little difference, which can be seen in the assembly quality metrics which yielded no significant differences between sequencing chemistries for the most part. The only exception was when assessing the number of indels per Mb, with LSK112 assemblies having fewer indels, suggesting greater accuracy (Fig. 3F). This aligns with previous findings, which show the LSK112 chemistry to have significantly higher average sequence accuracy of 98.34% compared to LSK109, which had an average sequence accuracy of 96.52% [34]. It is also noteworthy that the LSK112 chemistry claims to be able to generate up to 50% duplex data (https://nanoporetech.com/about-us/news/oxford-nanopore-tech-update-new-duplex-method-q30-nanopore-single-molecule-reads-0; accessed 11th October 2023), with quality scores exceeding ~ Q30, which could further enhance assembly accuracy beyond what was observed in this study.

Despite LSK112 performing better with accuracy of assembly, it appeared to create more fragmented assemblies, which can be seen in significant differences in the N50 and number of contigs within each assembly (Fig. 3E, H). This may be the result of insufficient sequencing depth [46], although read depth following rarefaction and filtering was similar between the two kits. However, as noted the read length for our LSK112 metagenomic libraries were consistently lower than for our LSK109 libraries (Table S4). Thus, the similar read depth seen between the chemistries represented a lower coverage and depth for the LSK112 libraries. The more fragmented assemblies seen for both chemistries could also be caused by similarities between the genomes (e.g., *S. enterica* and *E. coli* [13]), whereby assembly is more challenging due to problems binning similar reads [35]. Long-fragments are more likely to result in more complete assemblies, allowing for more obvious discrepancies between species and better resolution of complex regions, which has been shown to improve assembly completeness [9, 16]. Thus, the slightly shorter reads seen in the LSK112 data may account for the more-fragmented assemblies (Table S5 and Figure S6) due to fewer read overlaps and difficulties in read-binning. Discrepancies between the kits may also represent differences due to the flowcell used. The manufacturing process for flowcells and damage to the pores during transport, storage and use can impact on the number of pores available for sequencing. ONT recommend storage of flowcells for a maximum of 12 weeks, and offer replacements should the pore count drop below the warranty threshold in this time (https://nanoporetech.com/document/flow-cell-check; accessed 29th October 2024). Since the flowcell health can have a significant impact on the efficiency of data generation, it is possible that the R10.4 flowcell used for LSK112 sequencing showed decreased pore health compared to the R9.4.1 flowcell used for LSK109.

Other external factors may also affect sequencing efficiency. For example, ONT have recently identified an impact of light on in-run pore lifetime, with use of light shields resulting in a significantly greater improvement for R10.4.1 flowcells compared to R9.4.1 flowcells (https://nanoporetech.com/support/flow-cells/light-shields/why-do-i-need-to-put-a-light-shield-on-my-flow-cell; accessed 7th May 2024). Such a differential effect on sequencing throughput may explain discrepancies seen in read number, read length, and total mapped nucleotide bases between the LSK109 and LSK112 sequencing runs evident for each organism within the ZMC (Figure S6) despite both using the same DNA sample. Initial pore scans identified higher numbers of active pores for the LSK112 flowcell (1,292 pores) compared to the LSK109 flowcell (1,079 pores). However, the LSK112 flowcell saw a larger loss in pores over the 8-h run time (373 pores lost) compared to the LSK109 flowcell (77 pores lost). This indicates that differences in throughput were likely the result of more rapid pore deterioration for the LSK112 flowcell, potentially as a result of the light effects mentioned above. Technical replicates can be included by running libraries across multiple flowcells to address such technical errors, although this increases project costs and data analysis complexity. Overall, retainment of high-quality DNA when using kits featuring bead-beating technology can be hard, and there remains a trade-off between unbiased extraction and read-length. Thus, whilst all methods considered produced reasonable estimates for the sample diversity, the importance of genome completeness to the study being conducted must be considered when identifying the optimum DNA extraction approach.

The choice of DNA extraction methods, which can preferentially lyse bacteria with specific cellular structures, may introduce biases into the resulting data. Such biases are particularly important to consider when employing methodologies like adaptive sampling, as the initial composition and quality of the extracted DNA can significantly influence enrichment outcomes. In the first stage of this study, we compared a range of commonly used commercially available DNA extraction kits to compare the observed distribution of species identified from the well-characterised ZMC. These results show that DNA extraction kits that incorporate bead-beating methods for lysis of mixed microbial community samples provided the most accurate representation of the expected distribution, due to their ability to lyse organisms with tough cell walls such as Gram-positive bacteria. This is highlighted by the fact that underrepresentation of Gram-positive genera *Limosilactobacillus* and *Listeria* in most kits, both notoriously difficult to lyse [2], accounted for great variation from the expected composition (Fig. 1C). Although differences can also be seen within the kits that employed bead-beating (Fig. 1B), such as the overrepresentation of *Listeria* seen in the PS kit, this may be explained by the intensity of bead-beating due to differences in methodology. For example, MB and BM cluster more closely than other bead-beating kits and these utilised similar bead-beating methodologies using the FastPrep-24™ 5G bead beating grinder and lysis system (MP Biomedicals), compared to the PS and PF kits which were both lysed using a Vortex Adapter in either a 20 mL (PS) or 2 mL (PF) tube (Qiagen; Product No.: 13000-V1-12 or 13,000-V1-24) [65]. The larger surface area present during bead-beating for the PS kit may explain the larger proportion of *Listeria* compared to other kits. All kits were found to be significantly different from the expected composition which is likely due to differences in methodologies discussed resulting in larger under- and overrepresentations in the genera present (Fig. 1B, C).

Whilst bead-beating approaches provide a more complete picture of unknown mixed community bacterial samples, this may come at the cost of DNA integrity, with mechanical lysis approaches more likely to shear DNA than chemical lysis approaches. However, whilst bead-beating may significantly decrease the fragment length that can be achieved (typically < 10 Kb), long reads were still seen within the extracted DNA in this experiment (Figure S2; Table S2). This methodology remains popular for microbiome studies due to its reliability, cost-effectiveness, and efficiency, especially compared to more time-intensive high-molecular-weight DNA extraction protocols that often require ethanol precipitation. Consequently, the kits used in this study represent convenient and commonly utilised options within the microbiology community. Interestingly, despite lacking a bead-beating approach, resulting in the most biased composition compared to the ZMC, the AP methodology appeared to result in a more fragmented DNA yield (Figure S2). This has been seen previously with column-based kits [43]. Although other significant differences were seen between the PF and BM kits, the presence of only two replicates for the PF and PS groups may have impacted these results. Bray–Curtis dissimilarity showed the BM kit to be the most dissimilar to the ZMC composition, with the PF kit being the least dissimilar. Interestingly, similar values for AP and MB suggest that these biases are not entirely the result of bead-beating alone.

It should be noted that these analyses were performed using a mock community of known genera, whilst typically environmental metagenomic studies would demonstrate much higher diversity of organisms. Furthermore, the percentages of each organism within this sample do not represent the varied and often low abundance of many microorganisms in true mixed community samples (particularly for environmental samples). Despite this, these results provide a general view of the impact of DNA extraction on diversity and can help inform potential biases when diversity is previously understood, though applicability to real-life samples remains limited. To better assess detection limits and biases related to low-abundance organisms, additional standards such as the ZMC II (Zymo Research Corporation, California, USA; Product No.: D6310) could be used for future assessments. This community standard consists of the same organism composition as the ZMC, but with a logarithmic distribution of abundances, making it suitable for evaluating the lower limits of detection. As noted in its description, the ZMC is specifically designed for assessment of bias in DNA isolation across different samples. However, the ZMC II may offer valuable additional insights into bias affecting low-abundance organisms, which is more similar to what is seen in real-world samples. Low-abundance taxa often present the greatest challenges in metagenomic studies due to their susceptibility to loss during DNA extraction, amplification, or sequencing, as well as their dependency on high-sensitivity techniques for accurate detection. Without these low-abundance organisms, it is not possible to fully assess the detection limits or biases of the tested methods. Low-abundance organisms may be disproportionately lost during DNA extraction or sequencing, as their signals are more likely to fall below detection thresholds. Conversely, high-abundance organisms may dominate sequencing outputs, potentially skewing diversity metrics or masking the presence of less abundant taxa. These dynamics are largely absent in a mock community like ZMC, where all organisms are present in equal proportions, leading to an oversimplification of biases that would typically arise in real-world samples. Additionally, given the variation seen across extraction kits (Fig. 1B, C), future analyses may also benefit from evaluating contamination from the presence of potential extraction kit contamination (‘kitomes’), that have been found to be specific to differing extraction kits [56]. Kitomes are well known to contribute to differences in diversity between commercial DNA extraction kits, and may skew analyses, particularly those of high sensitivity. However, there are methodologies for remedying any unwanted effect of the kitome such as the introduction of appropriate negative controls during DNA extraction, and the application of bioinformatic tools such as GRIMER or MicrobIEM designed for contamination detection and removal [23, 45, 52].

The choice of sequencing chemistry is likely to make little difference to the contiguity or completeness of the resulting assembly, with the major improvement being increased accuracy and fewer indels. However, to ensure complete and high-depth assemblies are created, the use of longer sequencing runs to gather more data is likely to have a greater impact than the choice of sequencing chemistry. Indeed, improvements to the starting conditions such as higher initial proportions of the microbe of interest, or higher quality starting material with longer fragment lengths, are likely to be the most efficient way to produce improvements to metagenomic assemblies. For adaptive sampling, the loss of data throughput associated with this technique suggests that sequencing of shorter reads or the use of alternative software (as noted in other studies) may have a larger impact than the specific sequencing chemistry used, which showed no discernible difference in this study. Indeed, throughput limitations (as observed within this and other studies, and highlighted by ONT) may reduce the benefits of adaptive sampling, particularly when the goal extends beyond detection. Improvements to maximise on-target base-calling throughput have already been developed through software updates from ONT (such as the most recent update for MinKnow, v24.11.8), so users should use the most appropriate software available at the time of sequencing. However, careful consideration is needed when employing this approach to target extremely low-abundance organisms for whole genome sequencing. This limitation may warrant alternative strategies, such as depleting prominent known organisms to enrich for targets or regions of interest. However, this approach may be less applicable to environmental microbiology, where microbial compositions are often unknown and target organisms are frequently at very low abundances, making depletion challenging. Although Martin et al. [38] suggest adaptive sampling may improve the assembly of metagenomic genomes, the reduced throughput associated with this technique may mean it is not feasible for all cases. Adaptive sampling is therefore an incredibly powerful technique to support a wide range of applications in environmental and clinical microbiology, but its use and benefit should be carefully considered on a case-by-case basis.

## Conclusion

As demonstrated in this study, the DNA extraction methodology used when looking to explore the composition of taxa within mixed population microbial samples may impact on the resulting estimate of taxa present in the sample. In particular, methods incorporating bead-beating steps remain the most unbiased method for the extraction of hard-to-lyse organisms, with distributions in the well-characterised ZMC identified close to those expected (although still showing statistically significant differences). Additionally, differences in specific bead-beating methodologies may also impact resulting compositions. Such approaches may compromise fragment length due to shearing of the DNA, although reads of a reasonable length (> 10 Kb) may still be obtained for sequencing. However, this may make them less suitable for approaches where high molecular weight DNA is required (e.g., when the community contains species with long repetitive elements in their genomes). Whilst a clear impact of DNA extraction kit was identified, the sequencing chemistry used did not appear to impact the contiguity of the resulting assemblies from the metagenomic dataset. Minimal differences were identified when comparing results using identical DNA samples, with external factors affecting sequencing efficacy likely resulting in better or worse read length seen. Perhaps more critical is the volume of data available for the assembly of each metagenomic dataset, which consistently yielded high-quality contiguous genomes. However, whilst overall assembly metrics were similar between the two chemistries (Fig. 3), the improved base-calling quality associated with newer sequencing chemistries offers improved accuracy with respect to indels in comparison to a shared reference. This is also reflected following the use of adaptive sampling, which was shown to be an effective method for the enrichment of a species within a metagenomic sample, regardless of the sequencing chemistry used. However, due to potential impacts on throughput as a result of use of this approach, and practical considerations for the benefits of enrichment, use of this powerful new technique should be considered on a case-by-case basis. In conclusion, while extraction methodology has been shown to play an important role on the bias in the DNA composition obtained, sequencing chemistry used in this metagenomic context does not appear to make a substantial difference in genome completeness or to adaptive sampling enrichment for this microbial community sample.

## Supplementary Information

Supplementary material 1

## Data Availability

All data generated or analysed during this study are included in this published article and its supplementary information files. The raw sequencing data files used within the analyses are available from the National Center for Biotechnology Information (NCBI) Sequence Read Archive (SRA) under BioProjects PRJNA934869 and PRJNA934863. Publicly available data for the *S. cerevisiae* R64 genome was accessed from the NCBI RefSeq database (https://www.ncbi.nlm.nih.gov/assembly) using accession number GCF_000146045.2. This assembly contains 17 chromosomes with the following RefSeq accession numbers: NC_001133.9, NC_001134.8, NC_001135.5, NC_001136.10, NC_001137.3, NC_001138.5, NC_001139.9, NC_001140.6, NC_001141.2, NC_001142.9, NC_001143.9, NC_001144.5, NC_001145.3, NC_001146.8, NC_001147.6, NC_001148.4, NC_001224.1. Reference genome data for the ZymoBIOMICS control was obtained from Zymo Research through the following source: Zymo Research [67] ‘ZymoBIOMICS reference genomes (September 29, 2017) [Data set].’, *Zenodo* [Preprint]. Available at: https://zenodo.org/records/3935737. R Markdown code for the analyses performed within this paper are available from https://github.com/uopbioinformatics/Herbert_2025_Impact-of-Microbiological-Molecular-Methodologies-on-Adaptive-Sampling.

## Abbreviations

AP: AllPrep DNA/RNA mini kit
bp: Base pair
BM: FastDNA™ SPIN kit
BC: Bray-Curtis
Gb: Gigabase pair
Kb: Kilobase pair
MAG: Metagenomic assembled genome
MB: Quick DNA/RNA™ MagBead kit
ONT: Oxford Nanopore Technologies
PCoA: Principal coordinate analysis
PF: QIAamp PowerFecal pro DNA kit
PS: RNeasy PowerSoil DNA elution kit
ZMC: ZymoBIOMICS community standard
