# Supplementary material for: Impact of microbiological molecular methodologies on adaptive sampling using nanopore sequencing in metagenomic studies
Source: Environ Microbiome. 2025 May 5;20:47. doi: 10.1186/s40793-025-00704-7 (PMC12054170; doi:10.1186/s40793-025-00704-7)
Supplement: Supplementary file 1 — Supplementary material 1 [file 40793_2025_704_MOESM1_ESM.docx]

**Supplementary information**


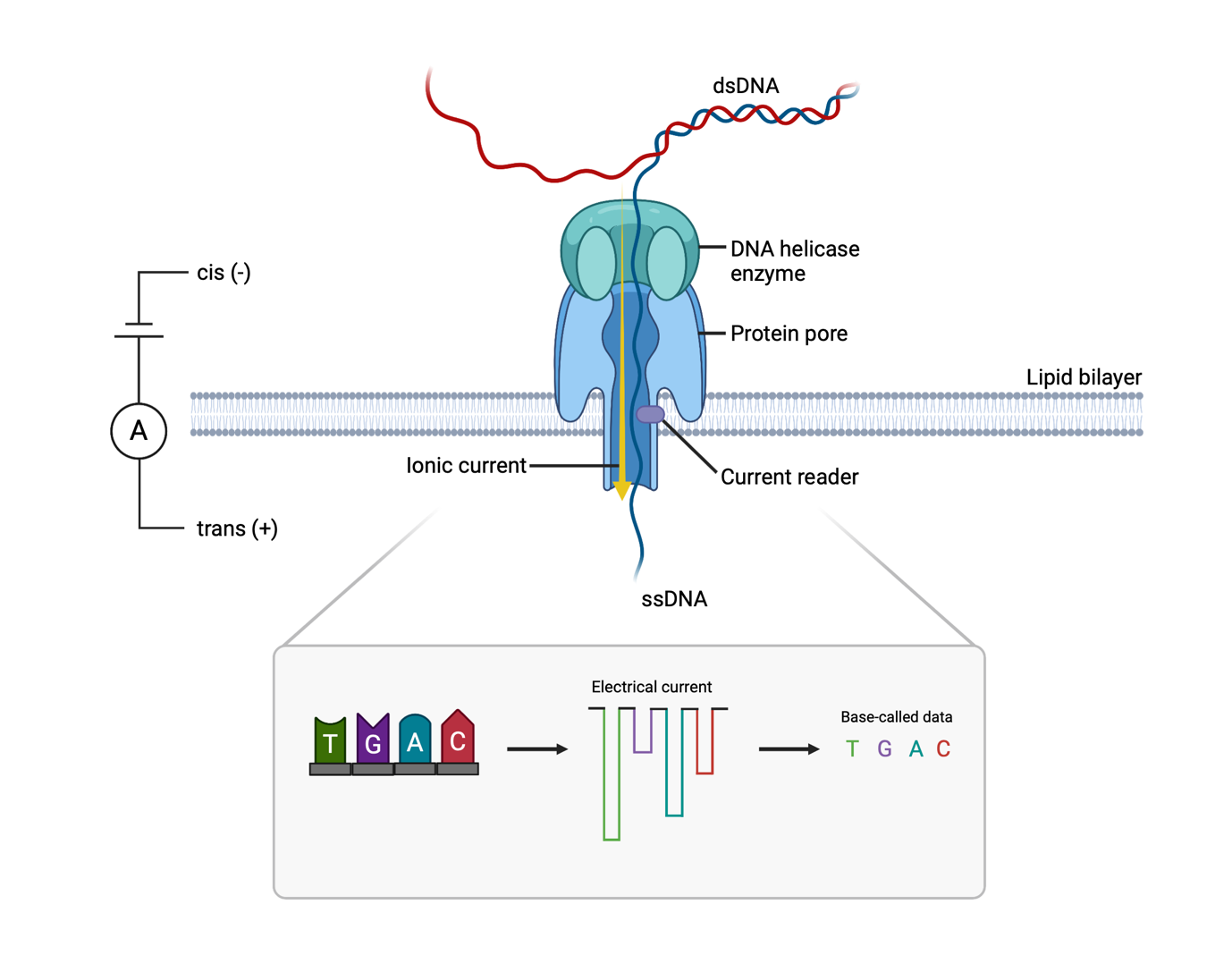


**Figure S1: Nanopore sequencing.** Schematic of ONT nanopore sequencing, showing dsDNA being unwound by helicase and the resulting ssDNA being translocated through the protein pore. Interruption of ionic flow across the pore as a result of nucleotide-specific structure and polarity is detected and transformed into DNA sequence information through a base-calling classifier for further bioinformatics analysis. Figure adapted with permission from (Beckett, Cook & Robson, 2021). Created with [BioRender.com](https://www.biorender.com/).

**Table S1:** **Composition of the ZymoBIOMICS Mock Community Standard.** The description of each of the 10 composite microorganisms of the ZymoBiomics Microbial Community Standard are shown, along with theoretical composition of extracted gDNA, genome copy number, 16S rRNA gene amplicon DNA, cell number, and the accession number for the strains contained within.

| **Species** | **Genus** | **Gram Stain** | **Genome Size (Mb)** | **Theoretical Composition of 16S rRNA (%)** | **Theoretical Composition of gDNA (%)** | **Theoretical Composition of Genome Copy Number (%)** | **Cell Number (%)** | **NRRL Accession No.** | **ATCC Accession No.** |
| --- | --- | --- | --- | --- | --- | --- | --- | --- | --- |
| *Pseudomonas aeruginosa* | *Pseudomonas* | - | 6.79 | 4.2 | 12 | 6.1 | 6.1 | B-3509 | 15442 |
| *Escherichia coli* | *Escherichia-Shigella* | - | 4.87 | 10.1 | 12 | 8.5 | 8.5 | B-1109 | - |
| *Salmonella enterica* | *Salmonella* | - | 4.76 | 10.4 | 12 | 8.7 | 8.8 | B-4212 | - |
| *Lactobacillus fermentum* | *Limosilactobacillus* | + | 1.90 | 18.4 | 12 | 21.6 | 21.9 | B-1840 | 14931 |
| *Enterococcus faecalis* | *Enterococcus* | + | 2.84 | 9.9 | 12 | 14.6 | 14.6 | B-537 | 7080 |
| *Staphylococcus aureus* | *Staphylococcus* | + | 2.73 | 15.5 | 12 | 15.2 | 15.3 | B-41012 | - |
| *Listeria monocytogenes* | *Listeria* | + | 2.99 | 14.1 | 12 | 13.9 | 13.9 | B-33116 | 19117 |
| *Bacillus subtilis* | *Bacillus* | + | 4.04 | 17.4 | 12 | 10.3 | 10.3 | B-354 | 6633 |
| *Saccharomyces cerevisiae* | *Saccharomyces* | Yeast | 12.10 | NA | 2 | 0.57 | 0.29 | Y-567 | 9763 |
| *Cryptococcus neoformans* | *Cryptococcus* | Yeast | 18.90 | NA | 2 | 0.37 | 0.18 | Y-2534 | - |

**Table S2:** **DNA extraction yield and quality.** Yield and quality statistics of DNA extracted from the ZymoBIOMICS Microbial Community Standard by each chosen extraction kit, as determined by 260/230 and 260/280 ratios determined by DS-11 FX/FX+ integrated UV-vis-spectrophotometer, with yield determined by Qubit fluorometric quantification using the high sensitivity double-stranded DNA assay kit (TL = too low to detect, NTC = negative control).

| **Commercial Kit** | **Abbreviation** | **Replicate** | **Volume Extracted (μL)** | **Yield (ng/μL)** | **260/230** | **260/280** | **Largest peak molecular weight and range (bp)** |
| --- | --- | --- | --- | --- | --- | --- | --- |
| AllPrep DNA/RNA Mini Kit | AP | 1 | 75 | 15.00 | 0.350 | 1.950 | 22,003  (5,046->60,000) |
|  |  | 2 | 75 | 15.70 | 0.500 | 1.880 | 20,641  (4,763->60,000) |
|  |  | 3 | 75 | 17.00 | 0.500 | 1.850 | 24,204  (5,064->60,000) |
|  |  | NTC | 0 | TL | - | - | - |
| RNeasy PowerSoil DNA Elution Kit | PS | 1 | 75 | 2.99 | 1.640 | 1.790 | 58,120  (52,097->60,000) |
|  |  | 2 | 75 | 1.99 | 1.570 | 1.260 | >60,000  (>60,000->60,000) |
|  |  | 3 | 75 | 15.30 | 1.950 | 1.650 | >60,000  (>60,000->60,000) |
|  |  | NTC | 0 | TL | - | - | - |
| QIAamp PowerFecal Pro DNA Kit | PF | 1 | 75 | 43.20 | 1.607 | 1.933 | 20,064  (5,689->60,000) |
|  |  | 2 | 75 | 36.80 | 2.008 | 1.906 | 17,925  (5,400->60,000) |
|  |  | 3 | 75 | 41.20 | 0.629 | 2.007 | 16,071  (5,179-56,744) |
|  |  | NTC | 0 | TL | - | - | - |
| FastDNA™ SPIN Kit | BM | 1 | 250 | 61.90 | 0.110 | 1.940 | 11,192  (4,496-30,746) |
|  |  | 2 | 250 | 72.90 | 0.160 | 1.902 | 11,398  (4,144-27,298) |
|  |  | 3 | 250 | 88.50 | 0.190 | 1.907 | 11,930  (4,198-36,473) |
|  |  | NTC | 0 | TL | - | - | - |
| Quick DNA/RNA™ MagBead Kit | MB | 1 | 75 | 16.1 | 2.739 | 1.716 | 14,903  (5,411-49,324) |
|  |  | 2 | 75 | 19.1 | 2.505 | 1.913 | 13,652  (5,111-37,582) |
|  |  | 3 | 75 | 19.5 | 2.804 | 1.952 | 13,193  (5,052-29,750) |
|  |  | NTC | 0 | TL | - | - | - |


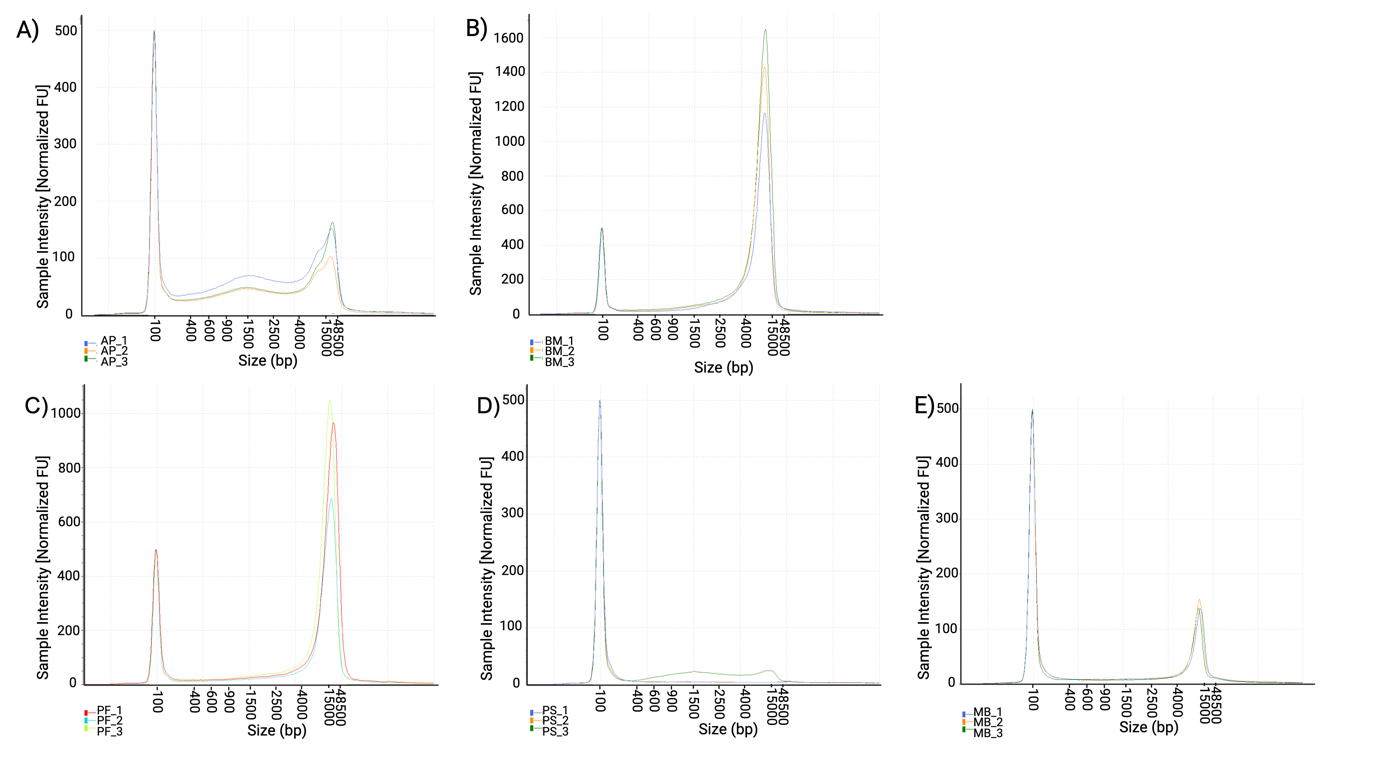


**Figure S2: DNA integrity.** Comparative electropherograms obtained through use of the 4150 TapeStation (Genomic DNA Assay) of each DNA extraction in triplicate using the four selected commercial kits: A) AllPrep DNA/RNA Mini Kit (AP); B) FastDNA™ SPIN Kit (BM); C) QIAamp PowerFecal Pro DNA Kit (PF); D) RNeasy PowerSoil DNA Elution Kit (PS); E) Quick DNA/RNA™ MagBead (MB).


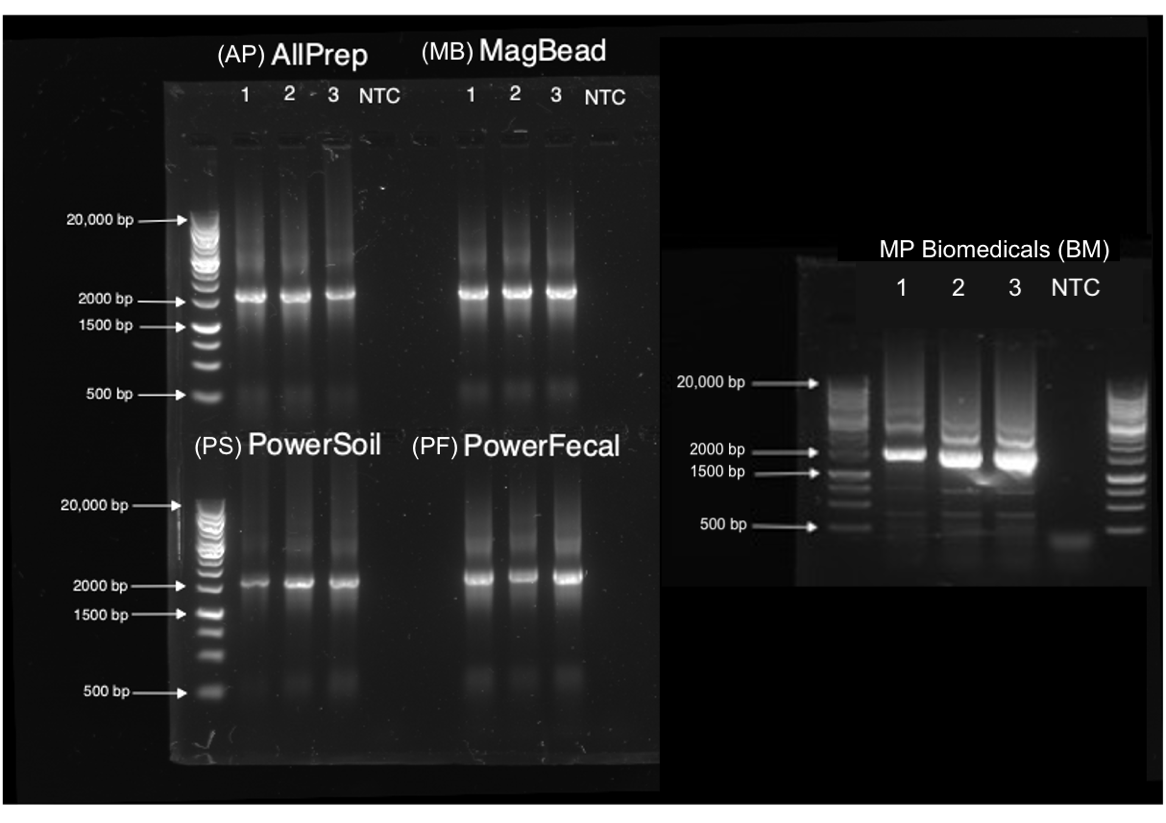


**Figure S3: 16S rRNA gene amplicon product QC.** Gel electrophoresis confirmation of full length 16S rRNA gene amplicon product from each DNA extraction in triplicate using the four selected commercial kits: AllPrep (AP); MagBead (MB); MP Biomedicals (BM); PowerSoil (PS); PowerFecal (PF), with negative extraction controls subject to the same treatment (NTC). Gels made at a percentage of 0.8% agarose and ran at 90 volts for 55 minutes, ladder used was the DNA 1 Kb plus ladder (Invitrogen™, Massachusetts, USA; Product No.: 10787018).

**Table S3: 16S rRNA gene sequencing yield.** The number of reads obtained from sequencing 16S rRNA gene amplified DNA from the ZMC for each DNA extraction kit tested in triplicate using sequencing chemistry LSK112.

| **Sample** | **Sequencing Chemistry** | **Replicate** | **No. Reads (Raw)** | **Median Quality Score**  **(Phred Q score)** | **Median Read Length (bp)** | **No. Reads (Phyloseq; before rarefaction)** |
| --- | --- | --- | --- | --- | --- | --- |
| BM | LSK112 | 1 | 305,482 | 16.4 | 1571 | 188,132 |
|  |  | 2 | 149,401 | 16.4 | 1581 | 91,859 |
|  |  | 3 | 274,358 | 16.3 | 1568 | 162,821 |
|  |  | NTC | 459 | 17.6 | 1493 | - |
| AP | LSK112 | 1 | 213,110 | 16.7 | 1407 | 157,276 |
|  |  | 2 | 72,894 | 17.1 | 1622 | 52,654 |
|  |  | 3 | 61,821 | 16.6 | 1618 | 42,850 |
|  |  | NTC | 190 | 17.4 | 1493 | - |
| PF | LSK112 | 1 | 2192 | 15.6 | 908 | 612 |
|  |  | 2 | 247,738 | 17.1 | 1627 | 192,526 |
|  |  | 3 | 75,762 | 17.0 | 1624 | 56,374 |
|  |  | NTC | 138 | 17.3 | 1503 | - |
| PS | LSK112 | 1 | 35,577 | 17.3 | 1625 | 26,903 |
|  |  | 2 | 132,315 | 17.3 | 1626 | 106,334 |
|  |  | 3 | 4133 | 16.2 | 1457 | 1,842 |
|  |  | NTC | 197 | 17.6 | 1498 | - |
| MB | LSK112 | 1 | 226,976 | 17.8 | 1625 | 181,315 |
|  |  | 2 | 200,058 | 17.2 | 1624 | 160,095 |
|  |  | 3 | 191,905 | 17.2 | 1624 | 151,362 |
|  |  | NTC | 125 | 16.7 | 1497 | - |


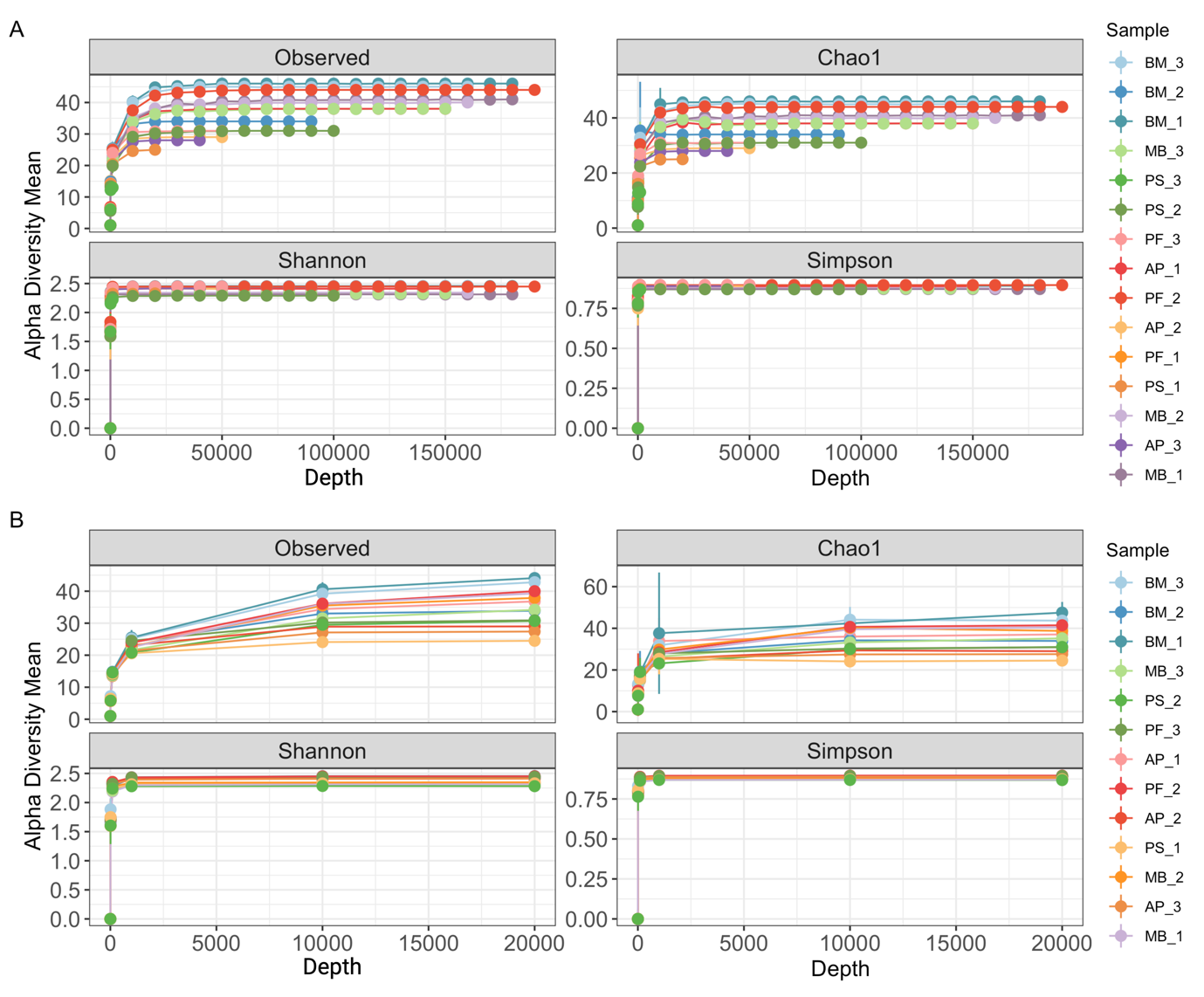


**Figure S4: 16S rRNA gene rarefaction analysis.** A) Rarefaction curve of 16S rRNA gene amplicon ZMC samples of the LSK112 sequencing run comparing different DNA extraction kits using observed, Shannon, Simpson and Chao1 alpha diversity indices. B) Rarefied samples of the LSK112 sequencing run comparing different DNA extraction kits using observed, Shannon, Simpson and Chao1 alpha diversity indices, rarefied to the sample with the lowest number of reads (>20,000) (PS_1; 26,903).


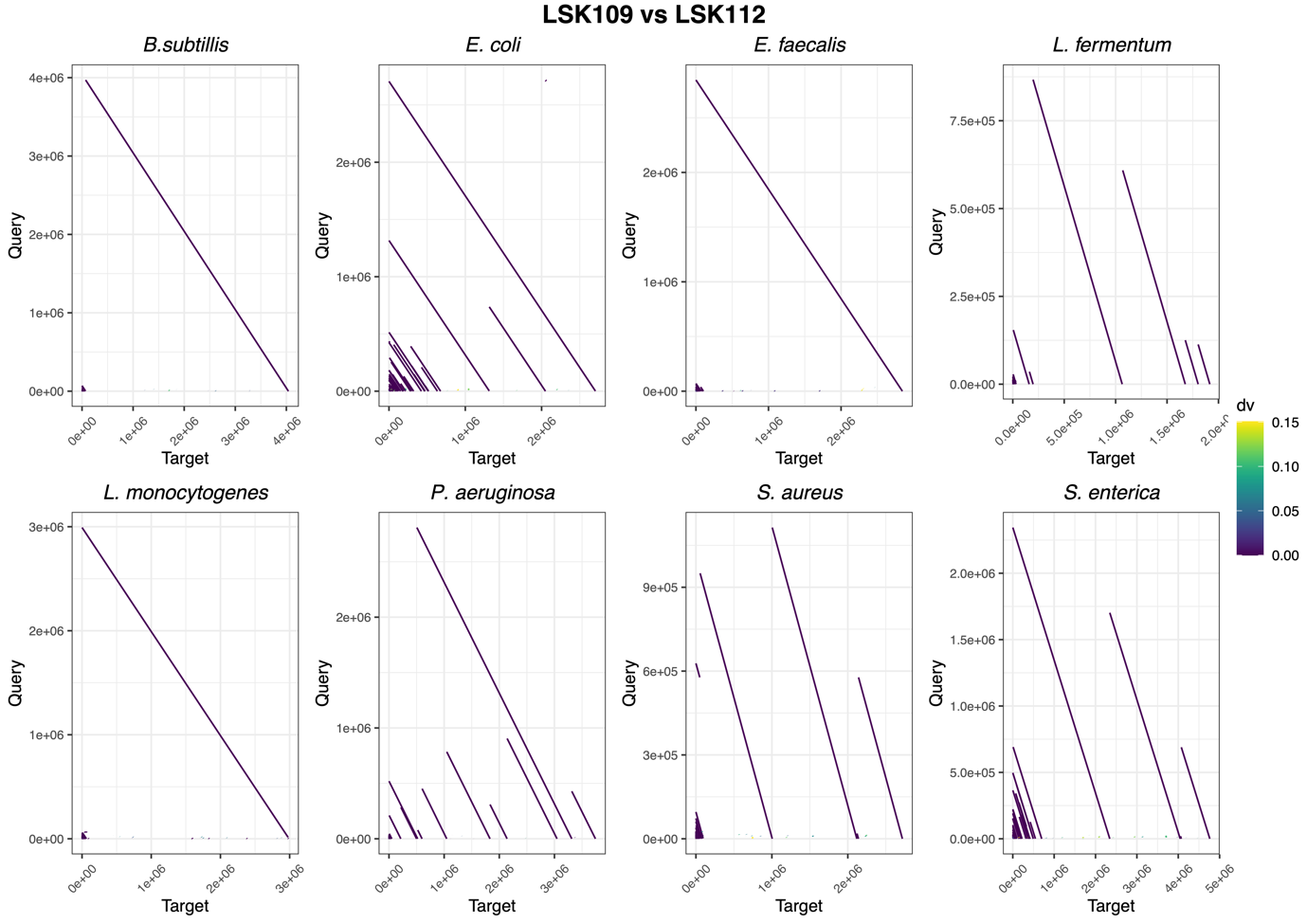


**Figure S5: Comparison of metagenomic assemblies between LSK109 and LSK112.** Dotplots of the assembled genomes of each expected bacterial species within the ZMC. Genomes assembled from LSK109 and LSK112 reads, aligned by divergence (dv) between the query (LSK109) and target (LSK112) (approximate per-base difference).

**Table S4: Metagenomic sequencing yield.** Yield data and values for full and adaptive metagenomic sequencing runs performed in triplicate using the LSK109 and LSK112 chemistry kits of ZMC DNA extracted using the BM kit, both before (raw) and after trimming (trim (full); Q=<7, <500 bp; trim (adaptive): Q=<7, <1000 bp, passed adaptive sampling) steps.

| **Sequencing**  **Chemistry** | **Library** | **Time Run (hours)** | **Replicate** | **Number of Reads** | | **Data (Megabases)** | | **Mean Read Quality**  **(Phred Q score)** | | **Mean Read Length (bp)** | | **Number of bases mapped to *S. cerevisiae*** | | **Percentage of bases out of total no. raw bases mapped to *S. cerevisiae* (%)** | |
| --- | --- | --- | --- | --- | --- | --- | --- | --- | --- | --- | --- | --- | --- | --- | --- |
|  |  |  |  | **Raw** | **Trim** | **Raw** | **Trim** | **Raw** | **Trim** | **Raw** | **Trim** | **Raw** | **Trim** | **Raw** | **Trim** |
| 109 | Full | 8 | 1 | 181,513 | 101,222 | 661.55 | 390.98 | 15.6 | 17.5 | 3,842.1 | 3,862.9 | 2,787,054 | 1,620,650 | - | 0.24 |
|  |  | 8 | 2 | 522,094 | 286,794 | 2003.05 | 1168.43 | 15.6 | 17.5 | 4,055.5 | 4,074.0 | 9,592,993 | 5,695,489 | - | 0.28 |
|  |  | 8 | 3 | 10,430 | 5,354 | 43.04 | 24.96 | 15.5 | 17.5 | 4,599.3 | 4,662.4 | 330,500 | 157,145 | - | 0.36 |
|  | Adaptive | 72 | 1 | 1,413,089 | 2,664 | 577.93 | 9.17 | 15.2 | 17.2 | 430.3 | 3,442.2 | - | - | - | - |
|  |  | 72 | 2 | 4,479,522 | 8,631 | 1893.87 | 31.43 | 15.1 | 17.2 | 446.7 | 3,641.3 | - | - | - | - |
|  |  | 72 | 3 | 87,981 | 142 | 34.57 | 0.71 | 15.1 | 17.0 | 443.8 | 5,003.9 | - | - | - | - |
|  |  | 8 | 1 | 545,263 | 1,188 | 233.61 | 4.08 | 14.0 | 16.5 | 428.4 | 3,433.9 | 8,019,109 | 4,079,494 | - | 1.74 |
|  |  | 8 | 2 | 1,663,439 | 3,738 | 739.29 | 14.03 | 13.9 | 16.5 | 444.4 | 3,753.0 | 27,268,150 | 14,028,753 | - | 1.89 |
|  |  | 8 | 3 | 32,006 | 61 | 14.16 | 0.33 | 13.9 | 16.1 | 442.5 | 5,353.2 | 685,166 | 326,544 | - | 2.30 |
| 112 | Full | 8 | 1 | 228,245 | 163,465 | 550.81 | 485.87 | 21.0 | 22.1 | 2,965.1 | 2,972.3 | 2,124,968 | 1,817,013 | - | 0.32 |
|  |  | 8 | 2 | 531,095 | 385,059 | 1081.76 | 953.32 | 21.2 | 22.4 | 2,475.5 | 2,475.8 | 4,902,040 | 4,277,183 | - | 0.39 |
|  |  | 8 | 3 | 16,633 | 11,942 | 41.18 | 36.46 | 21 | 22.2 | 1,546.5 | 3,053.4 | 181,712 | 162,480 | - | 0.39 |
|  | Adaptive | 72 | 1 | 1,413,089 | 1,233 | 226.89 | 3.49 | 20.8 | 22.3 | 444.3 | 2,833.5 | - | - | - | - |
|  |  | 72 | 2 | 4,479,522 | 3,152 | 499.46 | 7.93 | 20.8 | 22.6 | 419.7 | 2,514.8 | - | - | - | - |
|  |  | 72 | 3 | 87,981 | 117 | 16.70 | 0.34 | 21.0 | 22.6 | 444.4 | 2,887.7 | - | - | - | - |
|  |  | 8 | 1 | 312,301 | 776 | 138.76 | 2.30 | 17.6 | 20.3 | 444.3 | 2,964.6 | 2,822,291 | 2,300,500 | - | 1.65 |
|  |  | 8 | 2 | 721,524 | 1.943 | 304.69 | 4.94 | 17.6 | 20.3 | 422.3 | 2,542.9 | 6,168,271 | 4,934,001 | - | 1.61 |
|  |  | 8 | 3 | 23,102 | 78 | 10.16 | 0.21 | 17.7 | 20.6 | 439.9 | 2,741.2 | 269,199 | 213,813 | - | 2.10 |

**Table S5: Metagenome assembly statistics.** Genome completeness, relative scores of accuracy and assembly measurements of each expected bacteria within the ZMC, DNA obtained using the BM kit, sequenced using both LSK112 and LSK109 chemistries.

| **Species** | **Reference Genome Length (Mb)** | **Assembly Metrics** | | | | | | | | | | | | | | | | | |
| --- | --- | --- | --- | --- | --- | --- | --- | --- | --- | --- | --- | --- | --- | --- | --- | --- | --- | --- | --- |
|  |  | **Genome Fraction (%)** | | **No. Contigs** | | **N50 (Mb)** | | **Largest Contig (Mb)** | | **No. Indels** | | **Coverage** | | **Depth** | | **Mismatches per Mb** | | **BUSCO Completeness (%)** | |
|  |  | **LSK109** | **LSK112** | **LSK109** | **LSK112** | **LSK109** | **LSK112** | **LSK109** | **LSK112** | **LSK109** | **LSK112** | **LSK109** | **LSK112** | **LSK109** | **LSK112** | **LSK109** | **LSK112** | **LSK109** | **LSK112** |
| *Bacillus subtilis* | 4.22 | 99.95 | 99.77 | 21 | 28 | 4.04 | 3.97 | 4.04 | 3.97 | 353 | 90 | 100.00 | 99.99 | 18.15 | 25.18 | 17.5 | 17.1 | 100.00 | 100.00 |
| *Enterococcus faecalis* | 2.87 | 100.00 | 100.00 | 62 | 69 | 2.84 | 2.84 | 2.84 | 2.84 | 214 | 49 | 100.00 | 98.76 | 14.15 | 11.88 | 32.0 | 39.2 | 98.40 | 99.20 |
| *Escherichia coli* | 4.64 | 99.89 | 99.91 | 27 | 43 | 0.68 | 0.51 | 2.71 | 2.71 | 450 | 249 | 99.99 | 99.99 | 18.15 | 25.22 | 547.5 | 521.9 | 100.00 | 100.00 |
| *Lactobacillus fermentum* | 2.01 | 100.00 | 99.53 | 17 | 22 | 1.90 | 0.60 | 1.90 | 0.86 | 119 | 123 | 100.00 | 99.99 | 22.26 | 27.53 | 183.1 | 256.0 | 98.40 | 98.40 |
| *Listeria monocytogenes* | 2.94 | 99.93 | 99.99 | 76 | 83 | 2.98 | 2.99 | 2.98 | 2.99 | 141 | 68 | 99.99 | 99.91 | 14.68 | 11.68 | 40.1 | 31.4 | 99.10 | 100.00 |
| *Pseudomonas aeruginosa* | 6.26 | 99.97 | 99.78 | 99 | 107 | 3.04 | 0.78 | 3.74 | 2.80 | 256 | 43 | 99.99 | 99.99 | 16.02 | 17.49 | 91.3 | 40.1 | 100.00 | 100.00 |
| *Salmonella enterica* | 4.95 | 99.93 | 99.78 | 29 | 46 | 0.70 | 0.68 | 4.76 | 2.34 | 534 | 288 | 99.99 | 99.99 | 18.91 | 47.46 | 440.4 | 412.3 | 100.00 | 100.00 |
| *Staphylococcus aureus* | 2.82 | 99.57 | 99.41 | 55 | 79 | 2.71 | 0.62 | 2.71 | 1.11 | 146 | 22 | 100.00 | 99.99 | 12.87 | 11.59 | 40.1 | 11.4 | 100.00 | 100.00 |

**Table S6: Coverage and depth across chromosomes of *S. cerevisiae*.** Statistics of *S. cerevisiae* genome depth and coverage of reads gained from adaptive sampling using the LSK109 and LSK112 chemistry kits of ZMC DNA extracted using the BM kit, determined through use of Samtools depth.

| **Chromosome** | **Size (Kb)** | **Total no. reads aligned** | | **No. reference bases covered by reads** | | **Coverage (%)** | | **Mean depth** | |
| --- | --- | --- | --- | --- | --- | --- | --- | --- | --- |
|  |  | **LSK109** | **LSK112** | **LSK109** | **LSK112** | **LSK109** | **LSK112** | **LSK109** | **LSK112** |
| NC_001133.9 | 230.2 | 174 | 53 | 194,584 | 95,801 | 84.52 | 41.61 | 2.14 | 0.53 |
| NC_001134.8 | 813.2 | 694 | 291 | 742,082 | 463,840 | 91.26 | 57.04 | 2.86 | 0.85 |
| NC_001135.5 | 316.6 | 237 | 104 | 257,297 | 182,127 | 81.26 | 57.52 | 2.13 | 0.89 |
| NC_001136.10 | 1,530 | 1,222 | 513 | 1,393,752 | 841,386 | 90.98 | 54.92 | 2.94 | 0.92 |
| NC_001137.3 | 576.9 | 459 | 189 | 526,974 | 323,620 | 91.35 | 56.10 | 2.94 | 0.87 |
| NC_001138.5 | 270.2 | 205 | 71 | 229,524 | 98,742 | 84.96 | 36.55 | 2.93 | 0.47 |
| NC_001139.9 | 1,090 | 940 | 377 | 1,024,361 | 607,747 | 93.90 | 55.71 | 3.01 | 0.87 |
| NC_001140.6 | 562.6 | 430 | 162 | 501,078 | 279,895 | 89.06 | 49.75 | 2.69 | 0.73 |
| NC_001141.2 | 439.9 | 338 | 147 | 412,160 | 229,506 | 93.70 | 52.17 | 2.73 | 0.85 |
| NC_001142.9 | 745.8 | 639 | 228 | 673,171 | 435,275 | 90.27 | 58.37 | 3.02 | 0.85 |
| NC_001143.9 | 666.8 | 554 | 204 | 607,387 | 340,740 | 91.09 | 51.10 | 2.93 | 0.80 |
| NC_001144.5 | 1,080 | 1239 | 466 | 973,597 | 607,354 | 90.30 | 56.33 | 4.85 | 1.39 |
| NC_001145.3 | 924.4 | 781 | 266 | 853,765 | 503,483 | 92.36 | 54.46 | 3.02 | 0.77 |
| NC_001146.8 | 784.3 | 620 | 238 | 741,523 | 474,331 | 94.54 | 60.48 | 3.17 | 0.85 |
| NC_001147.6 | 1,090 | 863 | 337 | 1,008,163 | 575,434 | 92.38 | 52.73 | 2.91 | 0.82 |
| NC_001148.4 | 948.1 | 766 | 294 | 870,878 | 478,133 | 91.86 | 50.43 | 3.02 | 0.80 |
| NC_001224.1 | 85.8 | 957 | 333 | 79,970 | 79,386 | 93.23 | 92.55 | 21.71 | 6.44 |


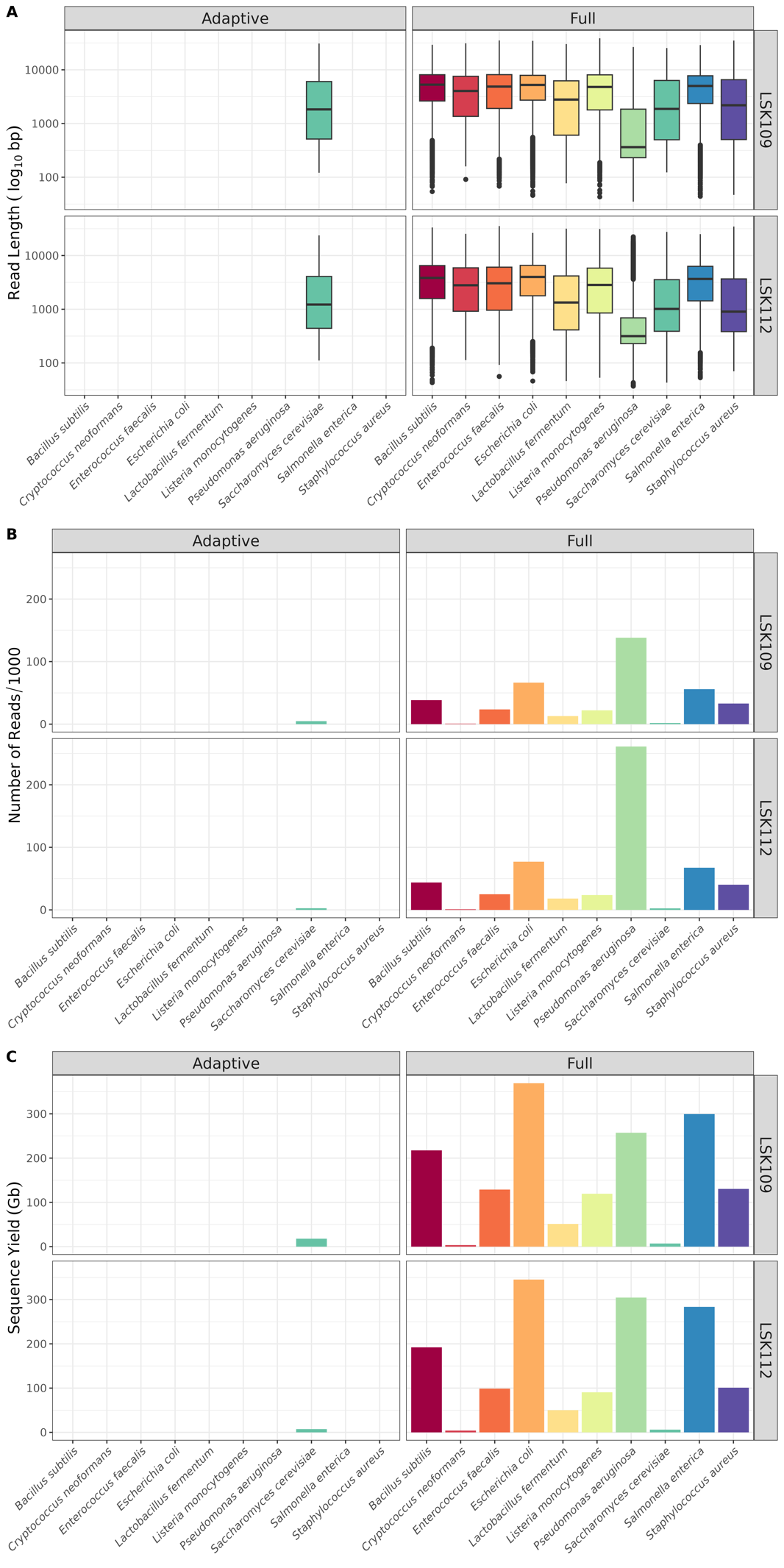


**Figure S6: Number of reads, read length, and data yield for each organism within the ZymoBIOMICS control for each sequencing chemistry.** Metrics for reads that were attributed to each of the ten species within the ZMC from full and adaptive sampling metagenomic sequencing runs, using either LSK109 or LSK112, showing; A) the distribution of the read lengths (log_10_ bp), B) the total number of reads (in thousands), and C) the total data yield (in Gb).
